# A20 enhances the migration and metastasis of gastric cancer cells by promoting occludin degradation

**DOI:** 10.1038/s41420-026-03082-2

**Published:** 2026-03-28

**Authors:** Yu-Ting Kuo, Hao-Chen Wang, Yan-Shen Shan

**Affiliations:** 1 Institute of Basic Medical Sciences, College of Medicine, National Cheng Kung University, Tainan, Taiwan, ROC; 2 Center of Comparative Medicine and Research, Innovation Headquarters, National Cheng Kung University, Tainan, Taiwan, ROC; 3 Institute of Clinical Medicine, College of Medicine, National Cheng Kung University, Tainan, Taiwan, ROC; 4 Division of General Surgery, Department of Surgery, National Cheng Kung University Hospital, College of Medicine, National Cheng Kung University, Tainan, Taiwan, ROC

**Keywords:** Tumour biomarkers, Gastrointestinal cancer

## Abstract

Chronic inflammation is a well-established risk factor in the development of gastric cancer (GC). Tumor necrosis factor α-induced protein 3 (TNFAIP3, also known as A20) is an inflammation-associated protein that functions as an oncogene in various cancers, but the role of A20 in GC progression remains unclear. In this study, clinical analyses revealed that elevated A20 expression in GC patients was significantly associated with increased tumor aggressiveness and metastatic potential. Functionally, A20 overexpression enhanced GC cell migration, whereas its knockdown suppressed this effect. Moreover, A20 promoted epithelial-mesenchymal transition and reduced the tight junction protein occludin. Mechanistically, A20 induced occludin endocytosis and lysosomal degradation via its ovarian tumor (OTU) domain. Pull-down assays revealed that A20 interacts with the migration-related protein RhoA, increasing its stability and thereby sustaining ROCK2 phosphorylation, which contributes to occludin degradation. PLA further showed that mutation of the OTU domain disrupted the interaction between A20 and RhoA in AGS cells, indicating the necessity of the OTU domain for this interaction. In conclusion, our findings demonstrate that A20 promotes GC cell migration by stabilizing RhoA and facilitating occludin degradation, underscoring A20 as a potential therapeutic target to inhibit GC metastasis.

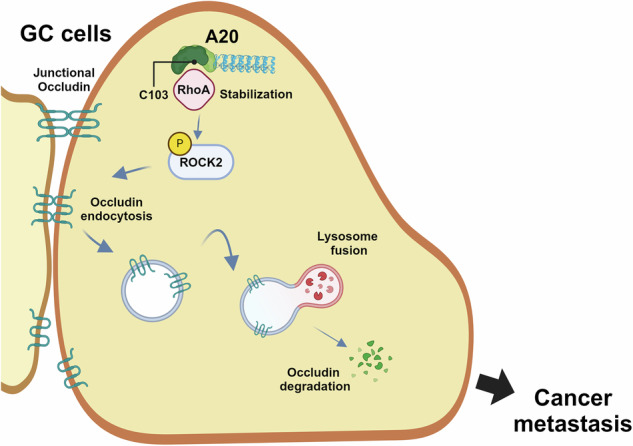

## Introduction

Gastric cancer (GC) is one of the deadliest malignancies worldwide, ranking 4th in cancer incidence and 5th in cancer-related mortality [1]. While early-stage GC is often associated with a favorable prognosis, more than 62% of patients are diagnosed with metastatic disease, which significantly worsens clinical outcomes [2]. Despite advancements in basic research and targeted therapies that have improved treatment efficacy, the prognosis for late-stage GC remains poor [3]. Therefore, unveiling the molecular mechanisms underlying GC progression and metastasis is critical for the development of effective therapeutic strategies.

Previous studies have demonstrated that protumor inflammation can activate oncogenic signaling pathways that facilitate cancer progression and metastasis [4, 5]. GC has been recognized as an inflammation-associated cancer [6, 7], but the specific mechanisms by which inflammatory factors drive GC remain to be fully elucidated. One key player in inflammation signaling is TNF-α-induced protein 3 (TNFAIP3, also known as A20), which is rapidly induced by NF-κB upon stimulation with cytokines or environmental stressors via binding to the *TNFAIP3* promoter [8]. A20 is traditionally recognized as a negative regulator of NF-κB signaling through targeting IκBα, thereby limiting inflammatory responses [9]. However, emerging evidence indicates that A20 exhibits context-dependent functions in cancer. In several malignancies, elevated A20 expression has been associated with tumor progression through NF-κB-independent mechanisms [10–13], suggesting that its role extends beyond immune regulation and may contribute to oncogenic processes.

Cancer cell migration is a prerequisite for metastasis, which involves disruption of cell-cell junctions, cytoskeletal rearrangement, extracellular matrix remodeling, and chemotactic responses [14, 15]. Additionally, epithelial-mesenchymal transition (EMT) is also regarded as a pivotal biological process in tumor metastasis [16]. The metastatic cascade is orchestrated by a complex network of extracellular and intracellular signaling events [17]. Recent studies have indicated that elevated A20 expression promotes cancer progression and metastasis across several tumor types. For instance, in melanoma, A20 fosters tumor growth, metastasis, and resistance to BRAF-targeted therapies [18]. A20 also inhibits the cell surface translocation of the “eat-me” signal calreticulin via suppression of stanniocalcin 1 degradation to facilitate immune evasion [11]. In breast cancer and GC, A20 has been shown to promote EMT and malignant transformation [19, 20]. Despite these findings, the precise molecular mechanisms by which A20 contributes to GC metastasis remain largely unclear. Moreover, due to the limited available clinical evidence, the clinicopathological significance of A20 expression and its prognostic value in GC patients have not been fully elucidated [20, 21]. In our preliminary screening aimed at identifying inflammation-associated regulators involved in GC aggressiveness, A20 emerged as a potential candidate molecule. To further evaluate its clinical relevance, we examined A20 expression in GC specimens obtained from National Cheng Kung University Hospital (NCKUH). Our preliminary analysis demonstrated that high A20 expression was significantly associated with tumor aggressiveness and metastatic potential.

Based on these observations, we investigated the functional role of A20 in GC progression. In the present study, we identify a novel mechanism by which A20 promotes GC cell migration through the degradation of occludin, a critical component of tight junctions. We demonstrate that A20 enhances GC cell migration by inducing endocytic lysosomal degradation of occludin, thereby disrupting cell-cell adhesion. Collectively, our findings establish A20 as a key regulator of GC metastasis and suggest that targeting the A20-mediated occludin degradation pathway may represent a promising therapeutic strategy to suppress GC progression and metastatic dissemination.

## Results

### A20 is upregulated in GC and promotes cell migration and metastasis

**Fig. 1:**
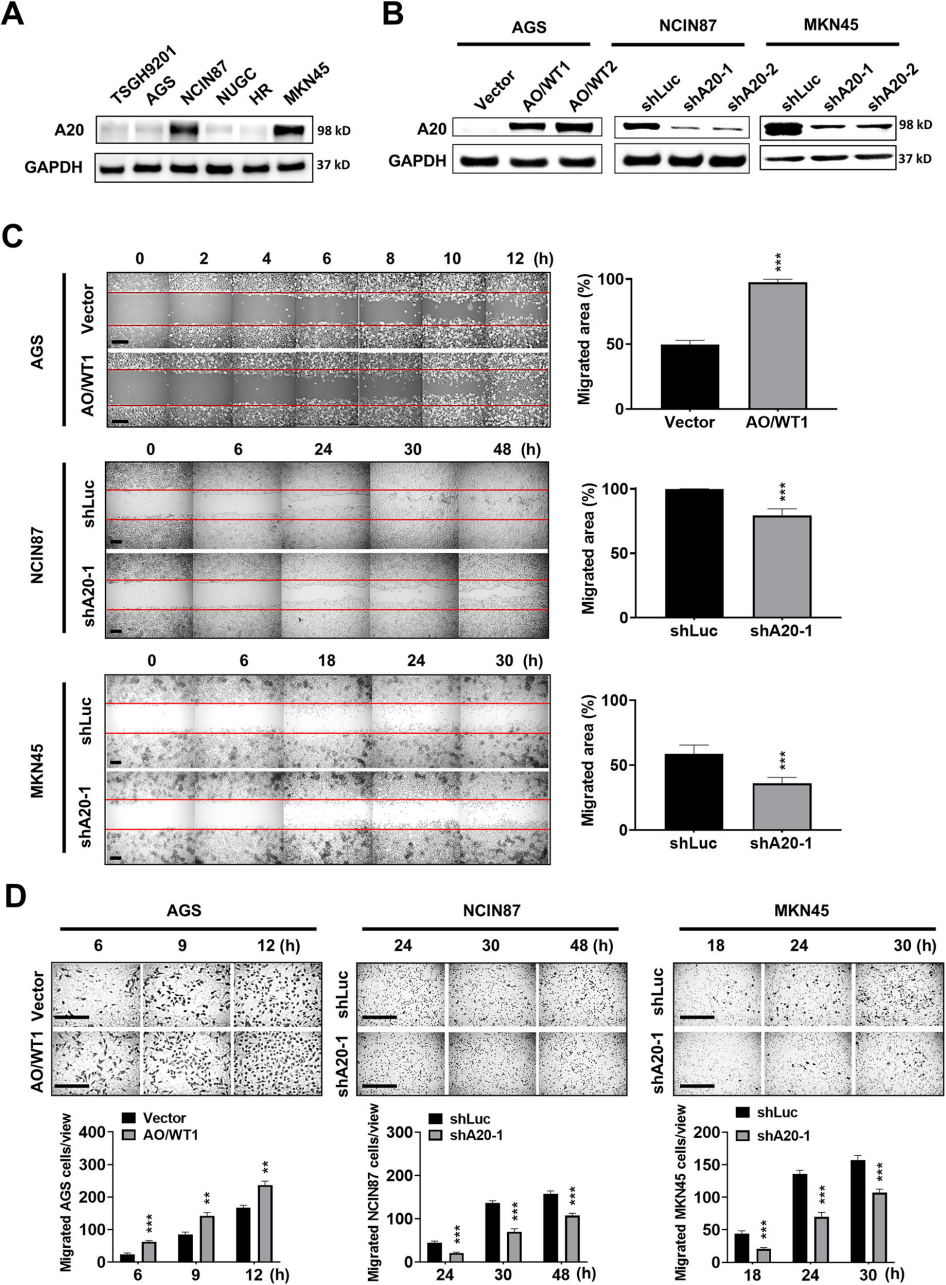
A20 enhances the migratory capacity of GC cells. **A** Western blot analysis of A20 expression across multiple gastric cancer (GC) cell lines. **B** Validation of A20 overexpression in AGS cells (AO/WT1 and AO/WT2) and A20 knockdown in NCIN87 and MKN45 cells (shA20-1 and shA20-2) by Western blotting. **C** Wound healing assays show that A20 overexpression (AO/WT1) increased migration in AGS cells, while A20 knockdown (shA20-1) reduced migration in NCIN87 and MKN45 cells. **D** Transwell migration assays confirmed that A20 overexpression promoted AGS cell migration, and A20 knockdown suppressed migration in NCIN87 and MKN45 cells. Data are presented as mean ± SEM from three independent experiments. **p < 0.01, ***p < 0.001.

We analyzed 122 GC patients with available tissue specimens to evaluate the clinical significance of A20 expression. Western blotting in 4 paired gastric tumor samples showed significantly higher A20 protein levels in tumor tissues compared with corresponding normal tissues (*p* = 0.0082, Supplementary Fig. S1A), consistent with IHC staining, which demonstrated higher A20 expression in both intestinal and diffuse type GC (Supplementary Fig. S1B). Patients were classified into four A20 expression grades (grades 1-4, *n* = 11, 30, 36, and 45, respectively, Supplementary Fig. S2A), and Kaplan–Meier survival analyses revealed that high A20 expression was associated with worse progression-free survival (PFS) and overall survival (OS) (both *p* < 0.001, Supplementary Fig. S2B). Demographic data of the 122 patients are shown in Supplementary Table S5. High A20 expression (grades 3–4) correlated with aggressive clinicopathological features, including male sex, subserosal invasion, lymph node metastasis, stage IV disease, perineural invasion, and tumor emboli, whereas no significant differences were observed for age ≥65 or Lauren classification (Supplementary Table S6 and Supplementary Fig. S3A, B). Notably, IHC also showed elevated A20 at the invasive front and in metastatic lymph nodes. Univariate Cox regression identified high A20 expression, advanced stage, subserosal invasion, lymph node metastasis, perineural invasion, tumor emboli, and Lauren classification as risk factors for poor PFS and OS. Multivariate analysis confirmed high A20 expression (grades 3–4) as an independent prognostic factor for both PFS (HR = 3.53, 95% CI: 1.684–7.400, *p* = 0.001) and OS (HR = 3.40, 95% CI: 1.608–7.169, *p* = 0.001), along with stage IV disease (Supplementary Table S7). Collectively, these data indicate that A20 is upregulated in GC and is associated with tumor aggressiveness and poor clinical outcomes. Besides, the datasets (GSE14210, GSE15459, GSE22377, GSE29272, GSE51105, and GSE62254) analyzed by the Kaplan-Meier (KM) Plotter (https://kmplot.com/analysis/) also revealed that high A20 mRNA expression contributed to lower survival rate of stage II and IV GC (Supplementary Fig. S4). We also performed Western blotting to screen A20 expression across various GC cell lines (Fig. 1A). To study the role of A20 in GC, we established A20 overexpressing AGS cell lines (AO/WT1 and AO/WT2) and A20 knockdown cell lines in NCIN87 and MKN45 cells (shA20-1 and shA20-2) (Fig. 1B). We found that expression levels of A20 were not associated with the proliferation of GC cells (Supplementary Fig. S5A). However, wound healing assays showed that A20 overexpression increased the migratory ability of AGS cells, while A20 knockdown reduced the migration of NCIN87 and MKN45 cells (Fig. 1C). Similar results were seen in transwell migration assays (Fig. 1D). Additionally, compared with control cells, A20-overexpressing AGS cells exhibited a mesenchymal-like phenotype, whereas A20 knockdown led to a shrunken, less spread morphology in MKN45 cells (Supplementary Fig. S5B). Collectively, these results indicate that A20 is able to increase GC cell migration.

**Fig. 2:**
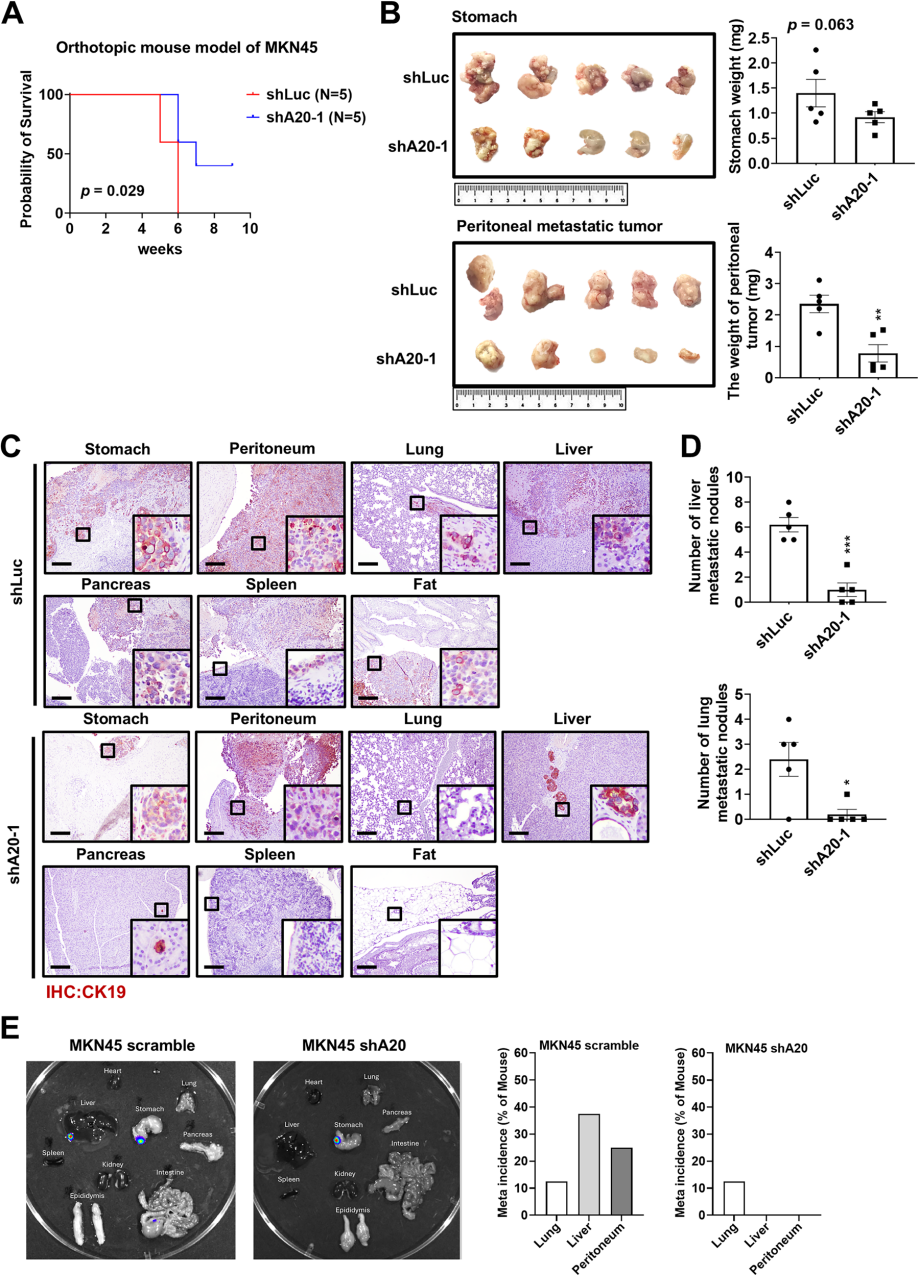
A20 promotes GC metastasis in vivo. **A** Kaplan–Meier survival analysis showed that A20 knockdown significantly prolonged survival of orthotopic MKN45 tumor-bearing mice (*p* = 0.029). **B** A20 knockdown did not significantly affect total stomach weight (*p* = 0.063) but markedly reduced peritoneal metastatic tumor burden. **C** IHC staining for cytokeratin 19 (CK19) revealed decreased distant organ metastasis in mice injected with A20 knockdown MKN45 cells compared to parental cells. **D** The number of metastatic tumor nodules was reduced per mouse following knockdown of A20. Data are presented as mean ± SEM (*n* = 5). **p < 0.01. **E** Metastatic tumors in distant organs were detected using IVIS imaging in the MKN45 scramble group (*n* = 8) and the MKN45 shA20 group (*n* = 8).

To further investigate whether A20 contributes to GC metastasis, GC cells were orthotopically inoculated into the stomachs of mice. A20 knockdown in MKN45 cells could increase survival compared to controls (*p* = 0.029) (Fig. 2A). Although the difference in stomach tumor weight between control and A20 knockdown groups was marginal (*p* = 0.061), A20 knockdown significantly reduced metastasis to the peritoneal cavity and distant organs (Fig. 2B–D, *p* < 0.01). IVIS imaging also confirmed that metastasis was reduced in MKN45 shA20 tumors carrying mice compared to MKN45 scramble tumors carrying mice (Fig. 2E). These results suggest that A20 exerts its oncogenic effects by promoting GC cell migration and metastasis.

### A20 downregulates the tight junction protein occludin in GC cells

Previous studies have reported the involvement of A20 in EMT [19, 20]. Accordingly, we sought to investigated the mechanism by which A20 regulates EMT in GC cells. In E-cadherin-negative AGS cells, A20 overexpression reduced the expression of occludin (Supplementary Fig. S6A). Conversely, A20 knockdown in NCIN87 cells increased occludin expression and decreased the levels of vimentin, snail, and twist1/2. Similarly, A20 knockdown in MKN45 cells also upregulated occludin and reduced N-cadherin, vimentin, snail, slug, and twist1/2 expression (Supplementary Fig. S6B). These results were further confirmed by qPCR analysis in AGS and MKN45 cells (Supplementary Fig. S6C).

**Fig. 3:**
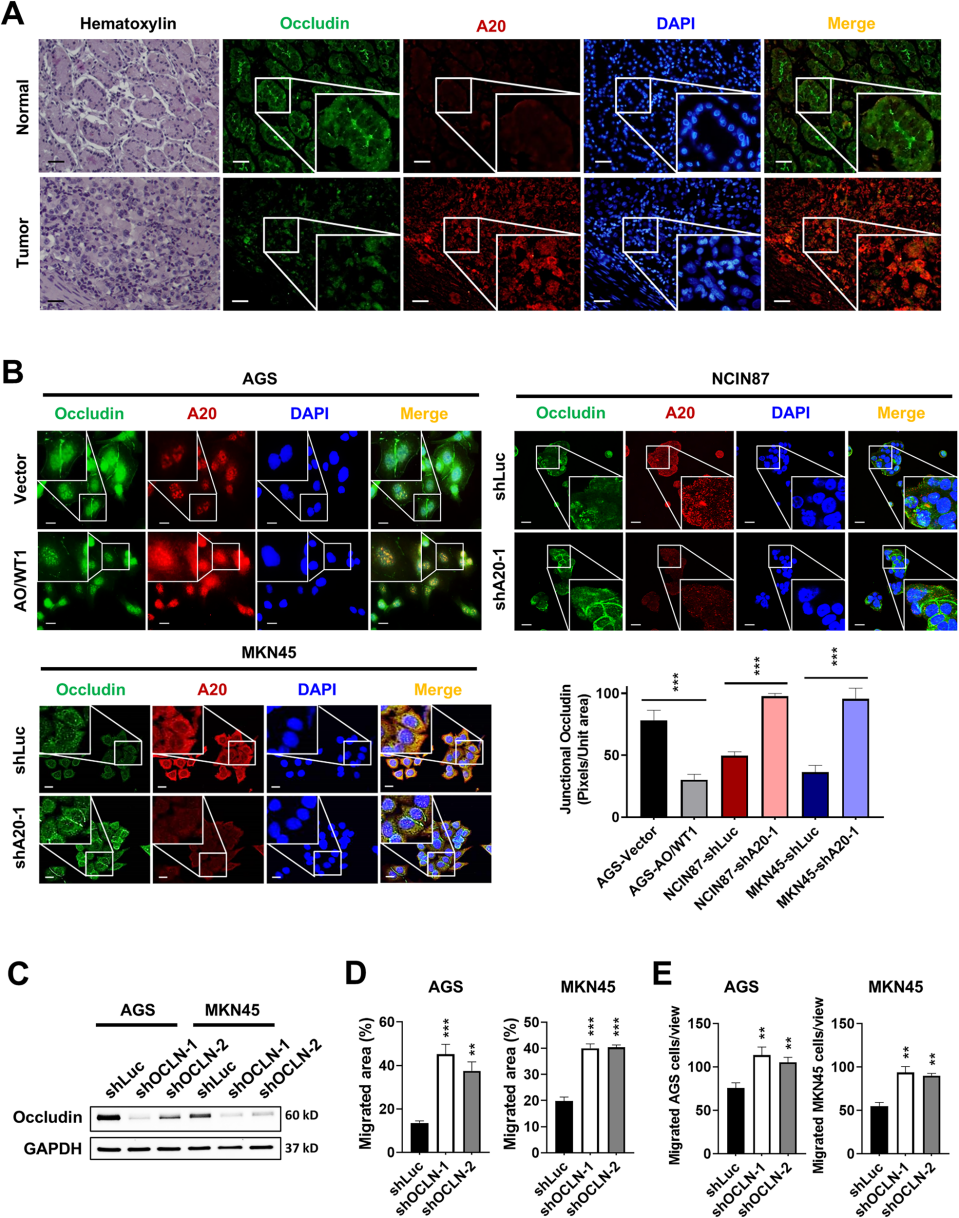
A20 induces occludin loss and disrupts tight junctions in GC tissue and cells. **A** Occludin expression was inversely correlated with A20 expression in human GC tissue samples. **B** A20 overexpression downregulated cell membrane-localized occludin in AGS cells, whereas A20 knockdown upregulated cell membrane-localized occludin in NCIN87 and MKN45 cells. **C** Knockdown efficiency of occludin in AGS and MKN45 cells was confirmed by Western blotting. **D**, **E**. Wound healing and transwell migration assays demonstrated that silencing occludin significantly enhanced the migratory capacity of AGS and MKN45 cells. Data are presented as mean ± SEM (*n* = 3 independent experiments). Significant differences are indicated (**p < 0.01, ***p < 0.001).

To identify potential mediators of A20-driven metastasis, we screened several tight junction and EMT-related proteins and found that occludin expression was most consistently and inversely correlated with A20 across GC cell lines. Importantly, occludin has a well-documented role in maintaining cell-cell junction integrity and limiting cancer cell migration [22–24]. Given this, we hypothesized that A20 promotes GC cell migration, at least in part, via downregulation of occludin. We further examined this relationship in clinical specimens. Immunofluorescence staining revealed an inverse correlation between A20 and occludin in GC tissues (Fig. 3A). In AGS cells, A20 overexpression (AO/WT1) induced occludin translocation to the cytoplasm and co-localization with A20, whereas A20 knockdown (shA20-1) maintained occludin localization at the cell membrane in NCIN87 and MKN45 cells (Fig. 3B). To evaluate the functional role of occludin in GC cell migration, we silenced occludin in AGS and MKN45 cells (Fig. 3C). Occludin depletion significantly enhanced the migratory capacity of both cell lines (Fig. 3D, E), supporting the hypothesis that A20 promotes GC cell migration, at least in part, via downregulation of occludin. Collectively, these findings indicate that A20 promotes EMT and facilitates the loss of occludin, thereby enhancing the migratory and metastatic potential of GC cells.

### A20 induces occludin degradation via endocytosis and lysosomal processing

**Fig. 4:**
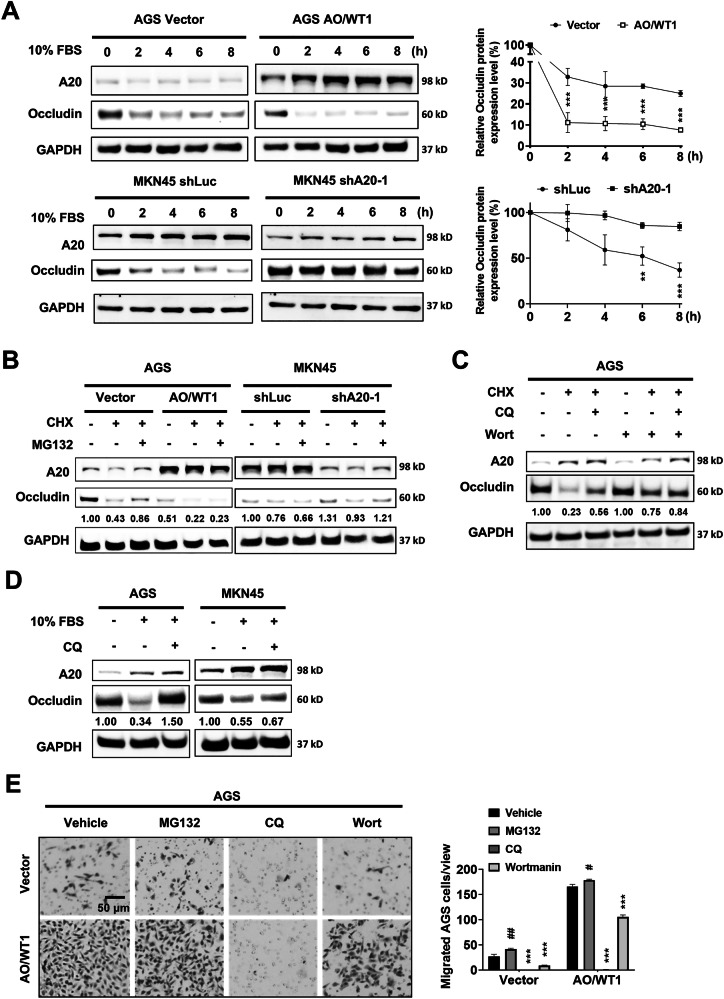
High A20 expression promotes occludin degradation via the lysosomal pathway. **A** Western blotting showed that occludin degradation was accelerated by A20 overexpression in AGS cells (AO/WT1), but occludin expression was preserved by A20 knockdown in MKN45 cells. Occludin expression was quantified relative to vector or shLuc controls (**p < 0.01, *p < 0.001). **B** Treatment with the proteasome inhibitor MG132 could not prevent occludin degradation in AO/WT1 AGS cells and MKN45 cells with high A20 expression. **C** Treatment with the lysosome inhibitor chloroquine (CQ) or the early endosome inhibitor Wortmannin (Wort) effectively inhibited occludin degradation in AGS cells. **D** A20-induced occludin degradation was suppressed after 24 h of CQ treatment in both AGS and MKN45 cells. **E** Transwell migration assays showed that treatment with CQ or Wort significantly inhibited A20-induced migration in AGS cells. Data are presented as mean ± SEM (*n* = 3 independent experiments). Significant upregulation: #*p* < 0.05, ##*p* < 0.01; significant downregulation: ***p < 0.001.

In response to environmental stimuli, occludin can translocate from the cell membrane to the cytoplasm, followed by endocytosis and subsequent proteasomal or lysosomal degradation [25]. Therefore, we sought to study whether A20 is involved in this process to regulate occludin degradation. After stimulation with FBS to induce EMT, occludin expression in AGS cells was decreased in a time-dependent manner, and this effect was further potentiated by A20 overexpression. Similarly, in MKN45 cells, occludin expression was also decreased over time, but knockdown of A20 attenuated this reduction (Fig. 4A).

To determine the proteolytic pathway in A20-mediated occludin degradation, we employed the proteasome inhibitor MG-132 in the presence of cycloheximide (CHX) that inhibits de novo protein synthesis. MG-132 restored occludin expression in vector control AGS cells and A20 knockdown MKN45 cells but had no effect in A20 overexpressing AGS cells and shLuc MKN45 cells, suggesting that A20 promotes occludin degradation through a proteasome-independent mechanism (Fig. 4B).

Given that lysosomal degradation contributes to occludin loss during tight junction disruption [26] and that the endocytosis inhibitor wortmannin can prevent tight-junction disassembly in epithelial cells [27], we investigated the role of endocytic and lysosomal pathways in A20-mediated occludin degradation. Treatment with the lysosome inhibitors chloroquine (CQ) as well as wortmannin in the presence of cycloheximide (CHX) could significantly inhibit occludin degradation in AGS cells (Fig. 4C). Under the stimulation of FBS, treatment with CQ restored occludin expression in both AGS and MKN45 cells (Fig. 4D). Moreover, CQ or wortmannin strongly counteracted A20 overexpression-induced cell migration in AGS cells (Fig. 4E), supporting the conclusion that A20 promotes GC cell migration by facilitating occludin degradation via enhanced endocytosis and lysosomal activity.

### The ovarian tumor (OTU) domain of A20 is critical to occludin endocytosis and lysosomal degradation

Previous research has demonstrated that the A20 OTU domain possesses deubiquitinating activity [28], and the ZnF4 domain and ZnF7 domain have catalytic activity as an E3 ligase [19, 29]. Mutations such as C103A in the OUT domain, C624A/C627A in the ZnF4 domain, and F770A/G771A in the ZnF7 domain can impair their enzymatic activity, indicating that these amino acid residues are critical for A20 biological functions. To determine which domain is essential for occludin degradation and cell migration, we generated A20 expression constructs harboring the C103A mutation in the OTU domain (OTU^mut^), C624A/C627A in the ZnF4 domain (ZnF4^mut^), and F770A/G771A in the ZnF7 domain (ZnF7^mut^) (Supplementary Fig. S7).

**Fig. 5:**
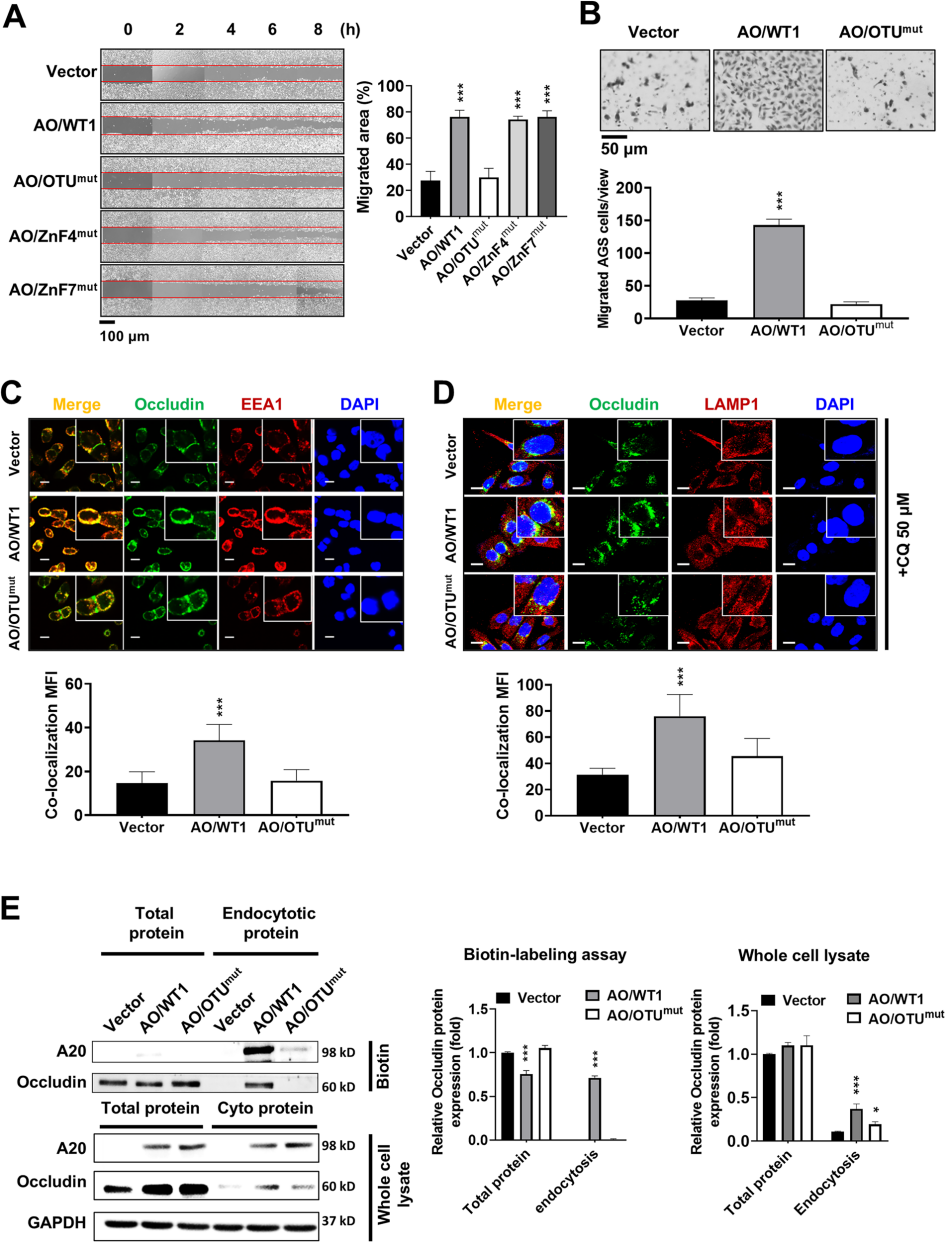
A20 regulates occludin endocytosis, translocation, and degradation via its OTU domain. **A** C103A mutation on the A20 OTU domain (OTU^mut^) reduced the migratory capacity of AGS cells (***P < 0.001.). **B** OTU^mut^ suppressed A20-induced migration in AGS cells (***p < 0.001.). **C** IF staining showed co-localization of occludin (green) with EEA1 (red) in vector control, AO/WT1, and AO/OTU^mut^ AGS cells. Scale bar = 10 μm. **D** Co-localization of occludin (green) with LAMP1 (red) was observed by IF staining. Scale bar = 10 μm. **E** Occludin endocytosis was inhibited in AO/OTU^mut^ AGS cells. Endocytic degradation of occludin was assessed by biotin-labeling endocytosis assay and membrane/cytosol fractionation. Data are presented as mean ± SEM (*n* = 3 independent experiments). Significant differences are indicated (*p < 0.05, ***p < 0.001).

Wound healing assays revealed that, in AGS cells, the OTU^mut^ construct blocked A20-induced cell migration, but Zn4^mut^ and Zn7^mut^ did not (Fig. 5A). This inhibitory effect of OTU^mut^ on cell migration was further confirmed by transwell migration assay (Fig. 5B). Because occludin degradation involves endocytosis and cytosolic trafficking, we examined the co-localization of occludin with early endosomes using IF staining for EEA1. The co-localization signal in AO/WT1 AGS cells was stronger than in AO/OTU^mut^ or vector control cells (Fig. 5C). Given our results that lysosomal degradation contributed to occludin loss, we performed dual IF staining for the lysosomal marker LAMP1 and occludin. The co-localization of occludin with LAMP1 was reduced in AO/OTU^mut^ cells compared to AO/WT1 AGS cells (Fig. 5D). Additionally, both biotin-based endocytosis assays and membrane/cytosol fractionation showed that occludin internalization was promoted in AO/WT1 AGS cells but was decreased in AO/OTU^mut^ cells (Fig. 5E). Taken together, these results demonstrate that the OTU domain of A20 is essential to promote occludin endocytosis and subsequent lysosomal degradation, thereby facilitating GC cell migration.

### A20-induced occludin degradation involves activation of the RhoA/ROCK2 signaling pathway

**Fig. 6:**
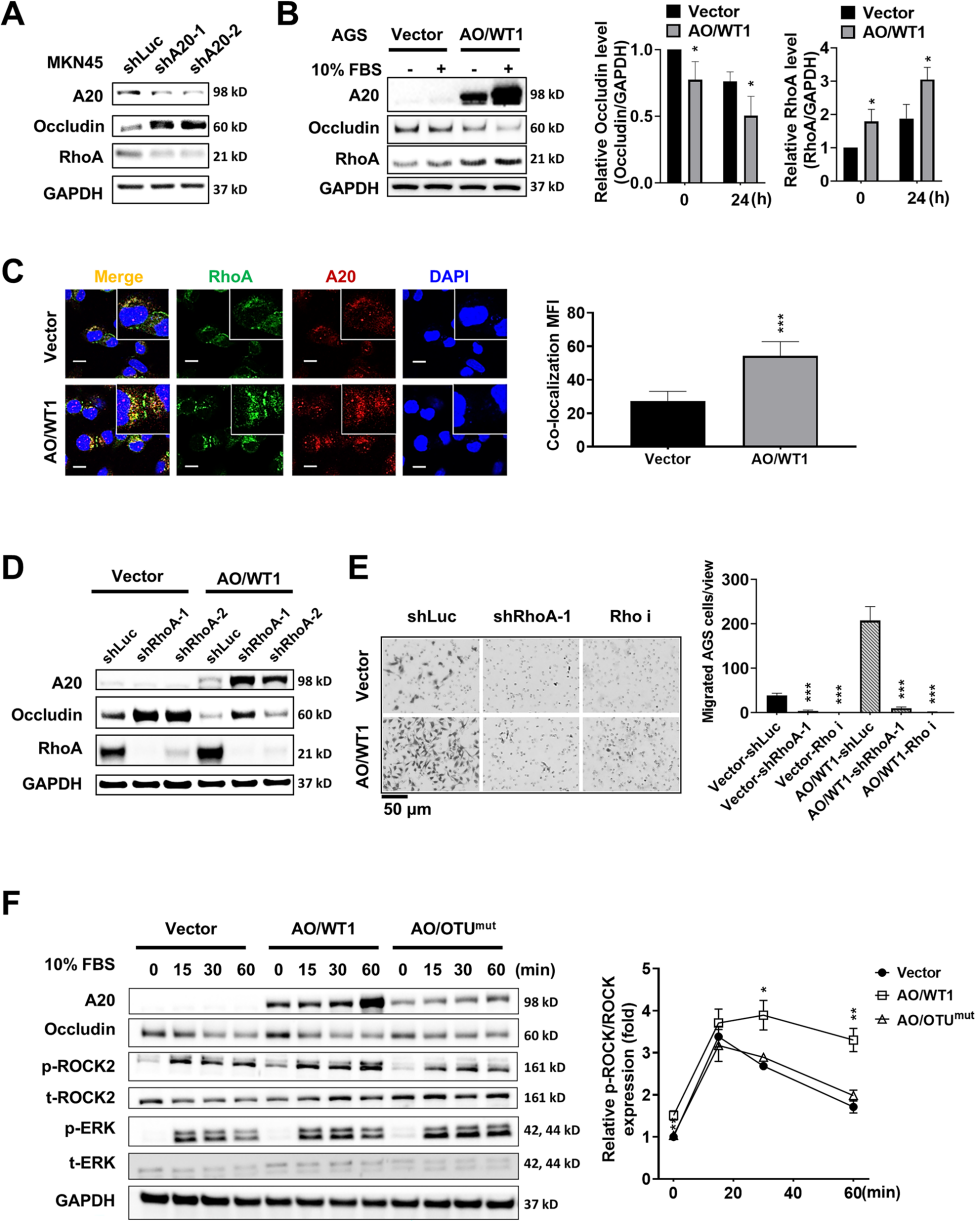
RhoA/ROCK2 signaling is involved in A20-induced migration and occludin degradation in GC cells. **A** Knockdown of A20 reduced RhoA expression in MKN45 cells. **B** Increased RhoA expression was detected in AO/WT1 AGS cells. **C** IF staining showed co-localization of A20 (red) with RhoA (green) in AGS cells. **D** RhoA knockdown upregulated occludin expression in both AO/WT1 and vector control AGS cells. **E** Knockdown of RhoA or treatment with the Rho inhibitor Rho i suppressed cell migration in both AO/WT1 and vector control AGS cells. **F** OTU^mut^ decreased ROCK2 phosphorylation without affecting ERK phosphorylation in AGS cells. Data are presented as mean ± SEM (*n* = 3 independent experiments). Statistically significant differences are indicated (*p < 0.05, **p < 0.01, ***p < 0.001).

To date, no studies have described how A20 regulates occludin degradation. To elucidate this mechanism, we conducted immunoprecipitation followed by mass spectrometry (IP-MS) to identify A20-interacting proteins potentially involved in occludin degradation and cancer metastasis. KEGG pathway analysis of proteins pulled down with Flag-tagged A20 revealed RhoA as a binding partner, known to be associated with tight junction assembly, actin cytoskeleton regulation, and endocytosis (Supplementary Fig. S8). In A20 knockdown MKN45 cells, RhoA expression was decreased and negatively correlated to occludin expression (Fig. 6A). Conversely, A20 overexpression enhanced RhoA expression 24 h after FBS stimulation and thus promoted AGS cell migration (Fig. 6B). IF staining further demonstrated increased expression and co-localization of A20 and RhoA in AO/WT1 AGS cells compared to vector control cells (Fig. 6C). RhoA knockdown increased occludin expression in both AO/WT1 and vector control AGS cells (Fig. 6D). Depletion of RhoA or inhibiting RhoA activity with a RhoA inhibitor (Rho i) could effectively decrease AGS cell migration (Fig. 6E). These results suggest that RhoA acts downstream of A20 to drive GC cell migration.

It has been reported that RhoA promotes occludin endocytosis by activating ERK or ROCK2 signaling [30, 31]. In our study, we observed that only ROCK2 phosphorylation was prolonged in AO/WT1 AGS cells, and this phosphorylation was diminished in AO/OTU^mut^ cells (Fig. 6F). Activation of RhoA/ROCK2 signaling promotes downstream F-actin assembly, a key indicator of cell migration. Phalloidin-Rhodamine staining revealed that AO/WT1 AGS cells exhibited increased expression of F-actin (red), which co-localized with endocytic occludin (green) (Supplementary Fig. S9A). To further assess the involvement of ROCK2 phosphorylation in occludin degradation, AGS and MKN45 cells were treated with an endocytosis inhibitor dynole and a ROCK2 inhibitor Y-27632. The inhibition of either endocytosis or ROCK2 effectively reduced occludin degradation in AGS and MKN45 cells (Supplementary Fig. S9B). Taken together, these results suggest that A20 promotes occludin degradation in GC cells through activation of RhoA/ROCK2 signaling.

### The OTU domain of A20 interacts with and stabilizes RhoA for occludin degradation

**Fig. 7:**
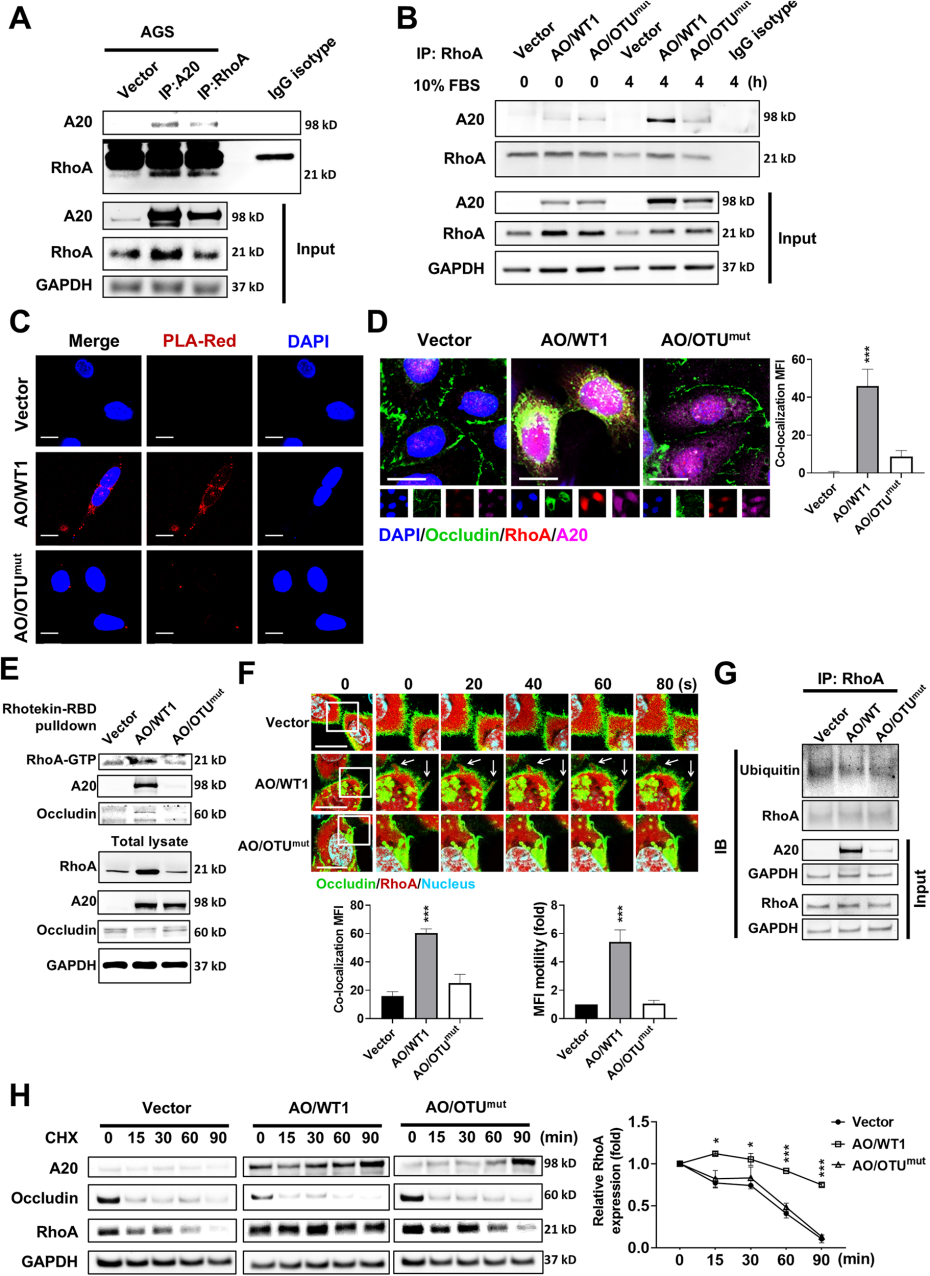
A20 interacts with and stabilizes RhoA through its OTU domain. **A** Co-IP confirmed the interaction between A20 and RhoA in AO/WT1 and vector control AGS cells. **B** RhoA pull-down assays revealed reduced A20-RhoA interaction in AO/OTU^mut^ AGS cells. **C** In situ IF staining showed that OTU^mut^ blocked A20-RhoA binding affinity in AGS cells. **D** Triple IF staining showed that co-localization of A20, RhoA, and endocytic occludin was disrupted in AO/OTU^mut^ AGS cells. **E** Pull-down of GTP-bound RhoA (GTP-RhoA) from vector control, AO/WT1, and AO/OTU^mut^ AGS lysates showed that OTU^mut^ impaired RhoA activation and occludin binding. **F** Time-lapse imaging showed that AO/WT1 AGS cells had greater dynamic co-localization and motility of cytosolic occludin-GFP and RhoA-OFP than vector control and AO/OTU^mut^ cells did. **G** RhoA pull-down assays showed that A20 overexpression reduced RhoA ubiquitination compared with vector control, whereas this effect was abolished in AO/OTU^mut^ AGS cells. **H** OTU^mut^ increased RhoA degradation in AGS cells. Data are presented as mean ± SEM (*n* = 3 independent experiments). Statistically significant differences are indicated (***p < 0.001).

To confirm the interaction between A20 and RhoA, we performed co-IP, which showed reciprocal binding between the two proteins (Fig. 7A). Their interaction was significantly weakened in AO/OTU^mut^ AGS cells after FBS stimulation, indicating that the C103A mutation in A20 OTU domain disrupts the binding of A20 with RhoA (Fig. 7B). The interaction was further validated in HEK293T cells co-transfected with A20 and RhoA (Supplementary Fig. S10). Proximity ligation assay (PLA) also showed a strong interaction between A20 and RhoA in AO/WT1 AGS cells but a marked reduction in AO/OTU^mut^ cells (Fig. 7C), accompanied by increased F-actin expression, suggesting enhanced cell motility. Triple IF staining demonstrated cytoplasmic co-localization of A20, RhoA, and occludin in AO/WT1 AGS and shLuc MKN45 cells, whereas occludin remained membrane-bound in vector, AO/OTU^mut,^ and shA20 cells (Fig. 7D and Supplementary Fig. S11A).

Rhotekin-RBD pull-down assays showed that A20 overexpression increased RhoA-GTP levels, while the OTU mutant impaired RhoA activity and disrupted its association with occludin (Fig. 7E). Live-cell imaging using occludin-GFP and RhoA-OFP confirmed enhanced cytosolic mobility only in AO/WT1 AGS cells (Fig. 7F and Supplementary Fig. S11B). It is well established that the OTU domain of A20 possesses deubiquitinating activity. Accordingly, we investigated whether A20 mediates ubiquitin editing of RhoA through its OTU domain. Our data showed that RhoA ubiquitination was reduced in AO/WT1 AGS cells, whereas this effect was reversed in AO/OTU^mut^ AGS cells, indicating that A20 regulates RhoA ubiquitination in an OTU domain-dependent manner (Fig. 7G). Collectively, these results demonstrate that A20 OTU domain facilitates occludin endocytosis and degradation by stabilizing and activating RhoA in GC cells. To determine whether the OTU domain of A20 is required for RhoA protein stabilization, GC cells were treated with CHX. After 90 min of CHX treatment, the degradation of RhoA was slower in AO/WT1 AGS cells than in vector control and AO/OTU^mut^ AGS cells (Fig. 7H). Overall, these results suggest that the OTU domain of A20 protein can stabilize RhoA, thereby promoting occludin degradation and enhancing the migratory capacity of GC cells.

## Discussion

This is the first study that investigated the association between expression of A20 and clinical outcome in GC. The result discovered that high expression of A20 in tumor tissue was an independent risk factor for disease recurrence and long-term survival. We also found that high expression of A20 was significantly correlated to advanced pathological features of GC, including subserosal invasion, lymph node metastasis, stage IV, presence of tumor emboli, and perineural invasion. We also identify a novel mechanism in which A20 facilitates GC cell migration through its OTU domain by inducing occludin internalization and subsequent lysosomal degradation. While A20 has been previously implicated in epithelial-mesenchymal transition (EMT) and tumor progression, our findings clarify how A20 contributes to tight junction disassembly, a key step in cancer cell dissemination.

Previous studies have associated A20 with the EMT in GC cells [20], which aligns with our findings that expression of EMT markers was affected by A20 knockdown. Among the EMT markers, occludin, a critical component of tight junctions, plays a central role in maintaining epithelial barrier integrity. Its depletion contributes to increased cellular permeability and is commonly observed during cancer metastasis [32]. Occludin has also been linked to gastric epithelial hyperplasia and inflammation in animal models [33], and to cytosolic redistribution in GC cells following H*. pylori* infection [34]. Our study confirms occludin as a tumor suppressor, as its silencing increased GC cell migration, and identifies A20 as a promoter of occludin degradation.

Although tight junction loss has been known as a facilitator of tumor invasion [35], the role of A20 in this process remains vague. In contrast to our findings, Kolodziej et al. reported that A20 maintained tight junctions in intestinal epithelial cells by preventing LPS-induced occludin loss via its N-terminal activity [36]. This discrepancy may be due to cell type specificity or different stimuli, suggesting the context-dependent regulatory roles of A20 in inflammation and cancer [10, 37]. Despite earlier evidence associating A20 with metastasis in inflammation-associated cancers [12, 19, 20], this is the first detailed demonstration of A20 driving GC cell migration by promoting lysosomal occludin degradation.

Previous studies have reported that A20 is localized to endocytic compartments and interacts with lysosome-associated proteins LAMP1 and LAPTM5 [38, 39]. In our study, biotin-labeling endocytosis assays revealed that A20 enhances occludin internalization, an important early event of GC cell migration. Pharmacological inhibition of lysosomes or endocytosis suppressed both occludin degradation and GC cell motility, indicating that targeting this pathway to prevent occludin degradation, for example, using CQ, might be a promising anti-metastatic strategy. Further investigation is needed to define the trafficking route and machinery that mediate the endocytic pathway of occludin.

RhoA is a small GTPase protein belonging to the Rho family, which plays a well-established role in cancer progression, including tight-junction breakdown, EMT induction, and increased migration [40–42]. Tight junction components like occludin, ZO-1, and claudins form a barrier limiting cell dissemination, which can be disrupted by RhoA signaling [43]. Blockade of the RhoA signaling pathway effectively suppresses cancer aggressiveness [44]. A meta-analysis by Nam et al. linked RhoA expression to advanced tumor stage and poor differentiation in GC [45]. Despite its relevance, little is known about the regulation of RhoA protein expression. Our results show that A20 can stabilize RhoA and consequently facilitate occludin degradation, indicating that A20 is a critical upstream regulator of RhoA in GC metastasis. Notably, RhoA silencing or inhibition could significantly restore occludin levels in A20 overexpressing cells.

Given the ubiquitin-editing enzymatic activity of A20, we explored the functional domains responsible for RhoA regulation. Prior studies have highlighted the oncogenic functions of its zinc finger domain in promoting EMT by mediating multiple mono-ubiquitin modifications of Snail [19], but our study found that the OTU deubiquitinase domain is also crucial for GC cell migration. We identified that A20 binds and stabilizes RhoA through its OTU domain. Mutation of the catalytic cysteine residue (C103A) in the OTU domain abrogated ROCK2 phosphorylation, suggesting that A20 acts as a regulator of the RhoA/ROCK2 pathway. However, the precise mechanism by which the OTU domain modulates RhoA stabilization and the downstream signaling pathways remains to be elucidated.

RhoA signaling has been reported to be essential for cytoskeletal reorganization, a prerequisite for cancer cell motility [46, 47]. Activated RhoA drives F-actin polymerization and reassembly of myosin and tubulin filaments, facilitating cell adhesion and migration [47, 48]. Live-cell imaging in this study showed co-localization and coordinated movement of A20, RhoA, and occludin, suggesting that A20 not only destabilizes tight junctions but also modulates cytoskeletal architecture to promote GC migration. Thus, A20, via its OTU domain, serves as a central modulator of both tight-junction integrity and cellular polarity. Notably, although our findings reveal the OTU domain of A20 as a potential therapeutic target, no specific inhibitors targeting this domain are currently available. Moreover, given A20’s well-established dual roles in immune regulation and inflammation, systemic inhibition may lead to unintended immunological consequences. Therefore, future therapeutic strategies will require the development of highly selective modulators or context-specific approaches to minimize adverse effects while preserving their physiological functions.

In conclusion, our study provided strong evidence that A20 stabilizes RhoA through its OTU domain to induce occludin internalization and lysosomal degradation, thus promoting GC cell migration and metastasis. This newly identified A20/RhoA/occludin signaling axis offers mechanistic insight into GC progression and highlights the A20 OTU domain as a promising therapeutic target against metastasis.

## Materials and methods

### Patients and specimens

The current study retrospectively reviewed the GC patients who underwent curative resection in NCKUH from 1998 January 1 to 2008 December 31. The clinical data, including gender, age, tumor staging, pathological characteristics, cancer recurrence and mortality, were obtained from electrical medical records. Tumor tissue specimens were obtained from tissue bank. The study protocol was approved by the Institutional Review Board of NCKUH.

### Immunohistochemistry

Formalin-fixed paraffin-embedded (FFPE) blocks of 122 GC specimens were cut into sections (5-μm thick). Sections were first heated at 60 °C for 30 min and then were deparaffinized in xylene and dehydrated in a graded ethanol series. After deparaffinization and dehydration, the slides were pretreated in 10 mM sodium citrate buffer (pH 6.0) in a microwave oven for 5 min at 95 °C for antigen retrieval. The endogenous peroxidase activity was quenched by a mixture of 0.3% hydrogen peroxide solution in 100% methanol for 30 min incubation. After washing with PBS buffer, the sections were incubated at 4 °C with rabbit anti-human A20 monoclonal antibody overnight (1: 100 dilution; Genetex, GTX62211). After washed with PBS buffer, the slides were treated with HRP labeled anti-rabbit IgG for 1 h followed by color development using 3, 3’-Diaminobenzidine (DAKO JAPAN Inc.). Finally, cell nucleus was counterstained by hematoxylin. A20 expression was scored by staining intensity (0% = 1; 0-25% = 2; 25-50% = 3; > 50% = 4) and staining area (0% = 1; 0-25% = 2; 25-50% = 3; > 50% = 4), and then the formula: √intensity*area, round the number was used to grade A20 staining, grade 1 = 1, *n* = 11 (9.0%); grade 2 = 2, *n* = 30 (24.6%); grade 3 = 3, *n* = 36 (29.5%); grade 4 = 4, *n* = 45 (36.9%).

### Cell line and culture conditions

Human GC cell lines AGS, SNU-1, MKN45, NCI-N87, and KATO III were obtained from the Bioresource Collection and Research Center (BCRC, Taiwan), while the HR cell line was acquired from the Cell Bank of the Clinical Medicine Research Center, College of Medicine, National Cheng Kung University Hospital (NCKUH). All cell lines were cultured in RPMI medium (Hyclone) supplemented with 10% fetal bovine serum (FBS; Gibco), 1% glutamine, and 1% antibiotic-antimycotic, and were incubated in a humidified atmosphere of 5% CO_2_ at 37 °C. The short tandem repeat (STR) analysis was used for cell line authentication.

### Transformation and transfection in GC cell lines

To establish stable transfected cell lines, GC cell lines were separately infected with the appropriate pLKO.1 lentiviral vectors containing *TNFAIP3* (TRCN0000050961 and TRCN0000218517), *RHOA* (TRCN0000047710 and TRCN0000047712), and non-target Luciferin short hairpin RNA (shRNA) vectors (TRCN0000072243) that were purchased from the National RNAi Core Facility, Academia Sinica, Taipei, Taiwan. Briefly, GC cells were grown overnight with 0.5 mL of viral supernatant containing 8 μg/mL polybrene in medium. Fresh medium containing 2 μg/mL puromycin was added the next day. Cells were maintained in the continuous presence of puromycin. Plasmids pCMV-flag (PS100001, Origene), pCMV-A20-Myc-flag (RC221337, Origene), occludin cDNA ORF Clone, Human, C-GFPSpark® tag (HG15134-ACG, SinoBiological), and RhoA cDNA ORF Clone, Human, C-OFPSpark® tag (HG12110-ACR, SinoBiological) were constructed and then were used to transfected AGS cells by Lipofectamin 3000. Cells were maintained in the continuous presence of G418 (500 μg/mL, A1720, Sigma-Aldrich) or Hygromycin B (200 μg/mL, 31282-04-9, Sigma-Aldrich). The knockdown and overexpression efficiencies were checked by Western blotting. The mutagenesis of A20 was performed using the QuikChange Lightning- Mutagenesis Kit (Stratagene, USA). The primers for A20 plasmid mutagenesis and Sanger sequencing are shown in Supplementary Tables S1 and S2.

### Transwell migration assay

Cell migration assays were performed using 24-well inserts (Falcon cell culture inserts, 8-μm pore size; BD Biosciences). In brief, the lower chamber was filled with 600 μL of growth medium, and 1 × 10^5^ cells seeded into the upper chamber containing 100 μL serum-free medium were incubated at 37 °C for 9-48 h (depending on cell lines). Then cells attaching to the upper side of the membrane were removed gently with a cotton swab and rinsed. Cells that migrated through the membrane and attached to the bottom membrane were fixed in methanol for 10 min at room temperature and were stained with hematoxylin (MERCK). The number of migrating cells was quantified by counting five independent symmetrical visual fields under the microscope.

### Wound healing assay

Culture-Inserts 2 Well (80209, ibidi) was placed on a 6-well plate, providing two cell culture reservoirs divided by a 500 µm wall. A total of 5 × 10^4^ cells in 100 μL of serum-free medium were seeded in each well of the insert. Once the cells reached confluence, the cell density was monitored under the microscope until a 100% optically confluent cell layer was confirmed. The Culture-Insert was then gently removed using sterile tweezers. The cell layer was washed with growth medium to remove cell debris and non-adherent cells. Subsequently, 2 mL of growth medium was added to each well, and the cell migration process was monitored by capturing images at multiple time points over the following h. The images were quantified and analyzed to determine the area covered by migrated cells.

### Protein degradation analysis

After GC cells were collected and the supernatant was removed, the cell pellet was resuspended in serum-free medium. A total of 1 × 10^6^ GC cells were then plated and incubated overnight at 37 °C in serum-free medium. For occludin dynamics analysis, the serum-free medium was replaced with medium containing 10% FBS and incubated for 0, 2, 4, 6 or 8 h. In RhoA degradation analysis, the serum-free medium was replaced with 10% FBS medium supplemented with cycloheximide (CHX, 20 μg/mL, 239763-M, Sigma-Aldrich) and incubated for 0, 15, 30, 60 or 90 min. All cell lysates were collected and stored at –20 °C until further analysis.

### Animal model

An orthotopic mouse model of human GC was used in this study. MKN45 cells (2 × 10^6^) were suspended in 20 uL of Matrigel and were orthotopically injected into the stomach wall of NOD-SCID mice. After tumor development for 4 weeks, the body weight and survival rate of the mice were monitored. At week 12, the mice were sacrificed for tumor collection. Primary tumors, organs, and metastatic lesions were harvested from each mouse and weighed for subsequent analysis. IVIS (In Vivo Imaging System) imaging was performed to monitor the formation of metastatic tumors.

### Real-time PCR

Total RNA was isolated from GC cell lines using TRIzol Reagent (Invitrogen, USA) and chloroform (Sigma, USA) and was reverse transcribed into cDNA. Expression of mRNA was measured using the ABI 7500 Fast Real-Time PCR System (ABI, USA). Results were normalized to GADPH using the 2^−ΔΔCT^ method. The primers used for mRNA expression analysis are listed in Supplementary Table S3.

### Western blotting analysis

Whole cell lysates were collected using RIPA lysis buffer, and protein concentrations were determined using the Pierce BCA Protein Assay Kit (Thermo Fisher). A mixture of 25 μg protein and 6× loading dye was heated at 100°C for 5 min and then placed on ice for an additional 5 min. Protein samples were loaded onto a 12% SDS-polyacrylamide gel for electrophoresis and subsequently transferred to a PVDF membrane (Millipore, USA). The membrane was blocked with 5% non-fat milk diluted in 0.05% TBST for 1 h at room temperature. Following blocking, the membrane was incubated with primary antibodies overnight at 4 °C and then with secondary antibodies for 1 h at room temperature. Protein signals were detected using Immobilon Western Chemiluminescent HRP Substrate (Millipore, USA) according to manufacturer’s instruction and were visualized using the iBright FL1000 Imaging System. The antibodies used to detect protein levels are listed in Supplementary Table S4.

### Immunofluorescence (IF) staining

GC cells were seeded onto chamber slides at a density of 5 × 10^4^ cells per well. After fixation with paraformaldehyde and permeabilization with Triton X-100, the samples were incubated with primary antibodies overnight at 4 °C, followed by incubation with fluorescent-conjugated secondary antibodies at room temperature for 1 h. F-actin was stained with Rhodamine Phalloidin (PHDR1, Cytoskeleton), and nuclei were stained with 4, 6-diamidino-2-phenylindole (DAPI). IF images were acquired using a Confocal Laser Scanning Microscope FV3000 (Olympus, Tokyo, Japan). Fluorescence signals were quantified using ImageJ software (NIH, USA).

### Pull-down immunoprecipitation (IP) and IP-mass spectrometry (IP-MS)

IP was performed using the Millipore Protein G Plus/Protein A Magnetic Beads (16-663, Millipore). Briefly, cell lysates from 5 × 10^6^ cells were incubated with anti-DDK (1 μg, 14793S, Cell Signaling) and anti-RhoA (1 μg, sc‑418, Santa Cruz) antibodies. The immunoprecipitated proteins were either analyzed by western blotting or submitted to LC-MS/MS protein identification (Biotools). GTP-bound RhoA was pulled down using Rhotekin-RBD agarose beads (BK036-5, Cytoskeleton) and detected by Western blotting.

### Statistical analysis

Statistical analysis was performed using GraphPad Prism 5.0 (GraphPad Software Inc) and SPSS Statistics 17.0 (IBM). Pearson’s chi-squared test and t-test were used to assess the significant correlations between A20 expression and clinicopathologic parameters. Kaplan–Meier was used to compare the survival curves of GC patients with high A20 expression and low A20 expression. Univariate and multivariate survival analyses of progression-free survival (PFS) and overall survival (OS) were performed by using Cox’s proportional hazards regression model. Multivariate analysis of variables was assessed by stepwise selection. Each experiment was performed at least three times independently under identical conditions. Data are expressed as the mean ± standard error of the mean (SEM). Statistical differences were analyzed using Student’s *t*-test and one-way ANOVA, as appropriate. Statistical significance was defined as follows: *** *p* < 0.001, ** *p* < 0.01, and * *P* < 0.05.

### Reporting summary

Further information on research design is available in the Nature Research Reporting Summary linked to this article.

## Supplementary information

A20 enhances the migration and metastasis of gastric cancer cells by promoting occludin degradation

reporting summary

raw data of western figure

Western raw data for some figures

## Data Availability

The datasets supporting the conclusions of this article are included within this article (and its supplementary information files).
