# Supplementary material for: A20 enhances the migration and metastasis of gastric cancer cells by promoting occludin degradation
Source: Cell Death Discov. 2026 Mar 28;12:206. doi: 10.1038/s41420-026-03082-2 (PMC13153315; doi:10.1038/s41420-026-03082-2)
Supplement: Supplementary file 1 — A20 enhances the migration and metastasis of gastric cancer cells by promoting occludin degradation [file 41420_2026_3082_MOESM1_ESM.docx]

**Supplementary data**

**A20 enhances migration and metastasis of gastric cancer cells by promoting occludin degradation**

Yu-Ting Kuo^1^, Hao-Chen Wang^2^, Yan-Shen Shan^2,3,4^

^1^Institute of Basic Medical Sciences, College of Medicine, National Cheng Kung University, Tainan, Taiwan, Republic of China (YTK e-mail: [brucekuo00@gmail.com](mailto:brucekuo00@gmail.com))

^2^Center of Comparative Medicine and Research, Innovation Headquarters, National Cheng Kung University, Tainan, Taiwan, Republic of China (HCW e-mail: [esr2wang@gmail.com](mailto:esr2wang@gmail.com); YSS e-mail: [ysshan@mail.ncku.edu.tw](mailto:ysshan@mail.ncku.edu.tw))

^3^Institute of Clinical Medicine, College of Medicine, National Cheng Kung University, Tainan, Taiwan, Republic of China

^4^Division of General Surgery, Department of Surgery, National Cheng Kung University Hospital, College of Medicine, National Cheng Kung University, Tainan, Taiwan, Republic of China

**Correspondence and Reprint to**

Yan-Shen Shan, M.D., Ph.D.

Distinguished Professor

Department of Surgery, National Cheng Kung University Hospital

Institute of Clinical Medicine, College of Medicine, National Cheng Kung University.

No. 35, Siaodong Rd. Tainan City 704017, Taiwan, Republic of China

Tel: 886-6-2353535 ext. 3116

Fax: 886-6-2758781

E-mail: [ysshan@mail.ncku.edu.tw](mailto:ysshan@mail.ncku.edu.tw)

**
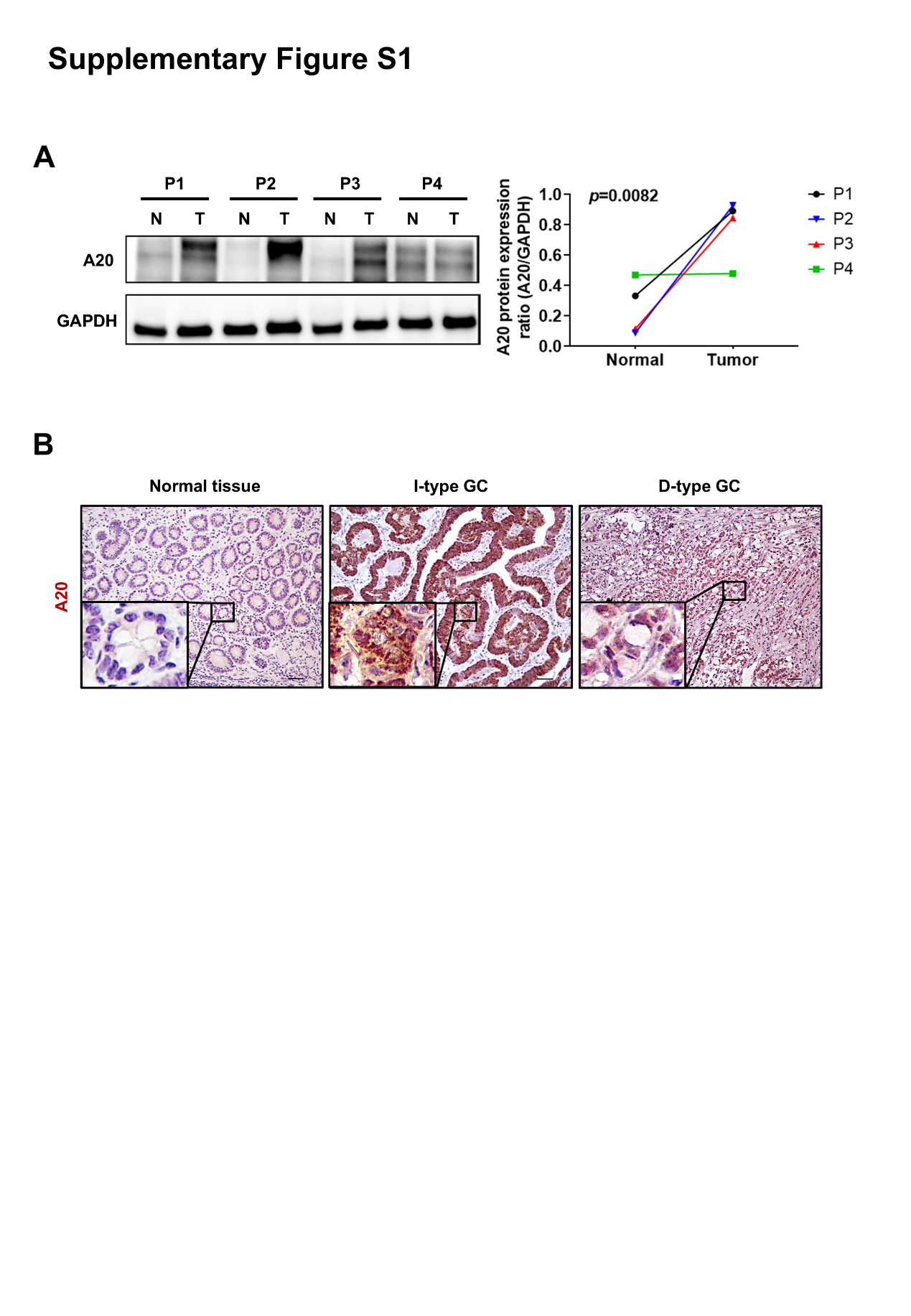
Supplementary Figure S1. A20 is highly expressed in human gastric cancer cells.**

**A.** A20 expression in gastric tumor tissues (T) and their corresponding normal tissues (N)*.* A20 expression was quantified and compared between the normal group and tumor group. *p* = 0.0082, unpaired, two-tailed Student’s t-tests. **B.** Representative IHC staining for A20 in normal gastric tissue, intestinal-type (I-type) gastric tumor tissue, and diffuse-type (D-type) gastric tumor tissue.


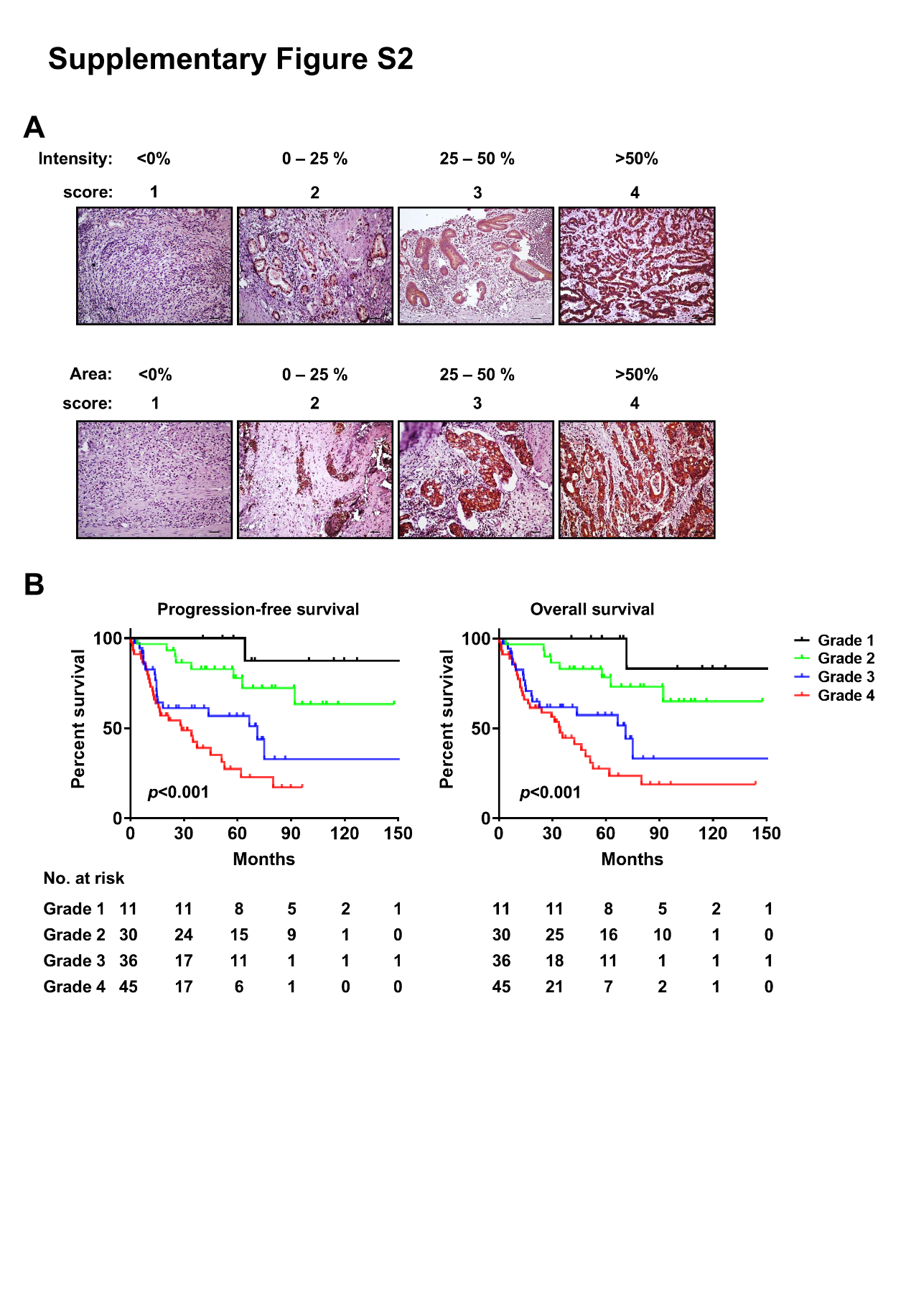


**Supplementary Figure S2. High expression of A20 negatively correlated to GC patient’s survival.**

**A.** IHC staining for A20 was performed in 122 gastric tumors and was scored by staining intensity and percentage. A20 expression was graded by the formula $\sqrt{intensity*area}$ as 1-4. **B.** The correlation of A20 levels with progression-free survival (PFS) and overall survival (OS) of GC patients was determined by Kaplan-Meier survival analysis. High A20 expression was strongly correlated to poor PFS (p < 0.001) and OS (p < 0.001) rate in GC patients. The numbers at risk were shown at 30-month intervals.


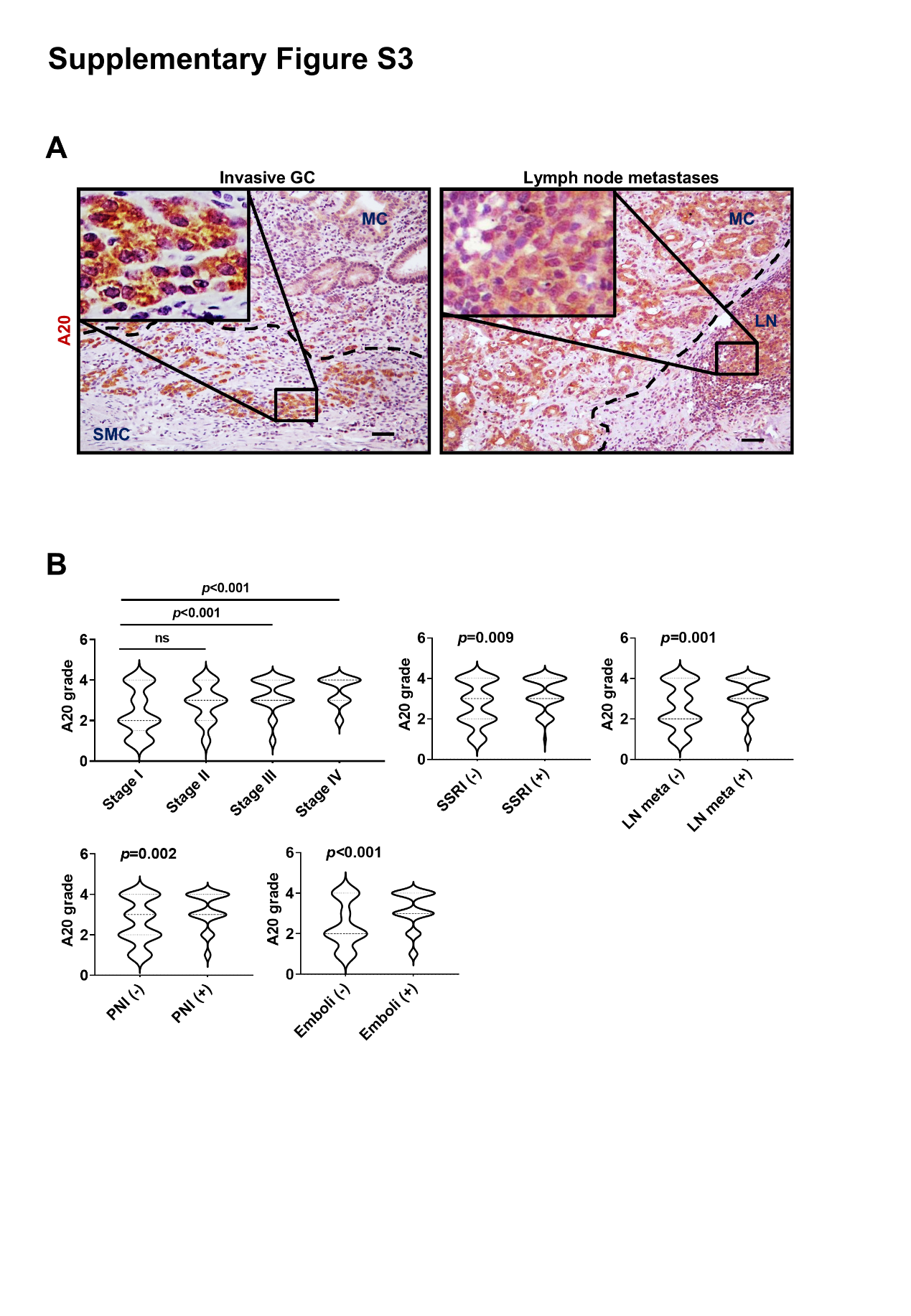


**Supplementary Figure S3. High A20 expression contributed to GC aggressiveness and metastasis.**

**A.** IHC staining for A20 was performed in gastric tumors and metastases. (MC: mucosa; SMC: submucosa; LN: lymph node; scale bar = 100 mm). **B.** A20 expression positively correlated to clinicopathologic characteristics including stage III (*p* < 0.001), stage IV (*p* < 0.001), subserosal invasion (SSRI, *p* = 0.0093), lymph node metastasis (LNM, *p* = 0.001), perineural invasion (PNI, *p* = 0.002), and tumor emboli (*p* < 0.001) in GC patients from NCKUH (N=122).


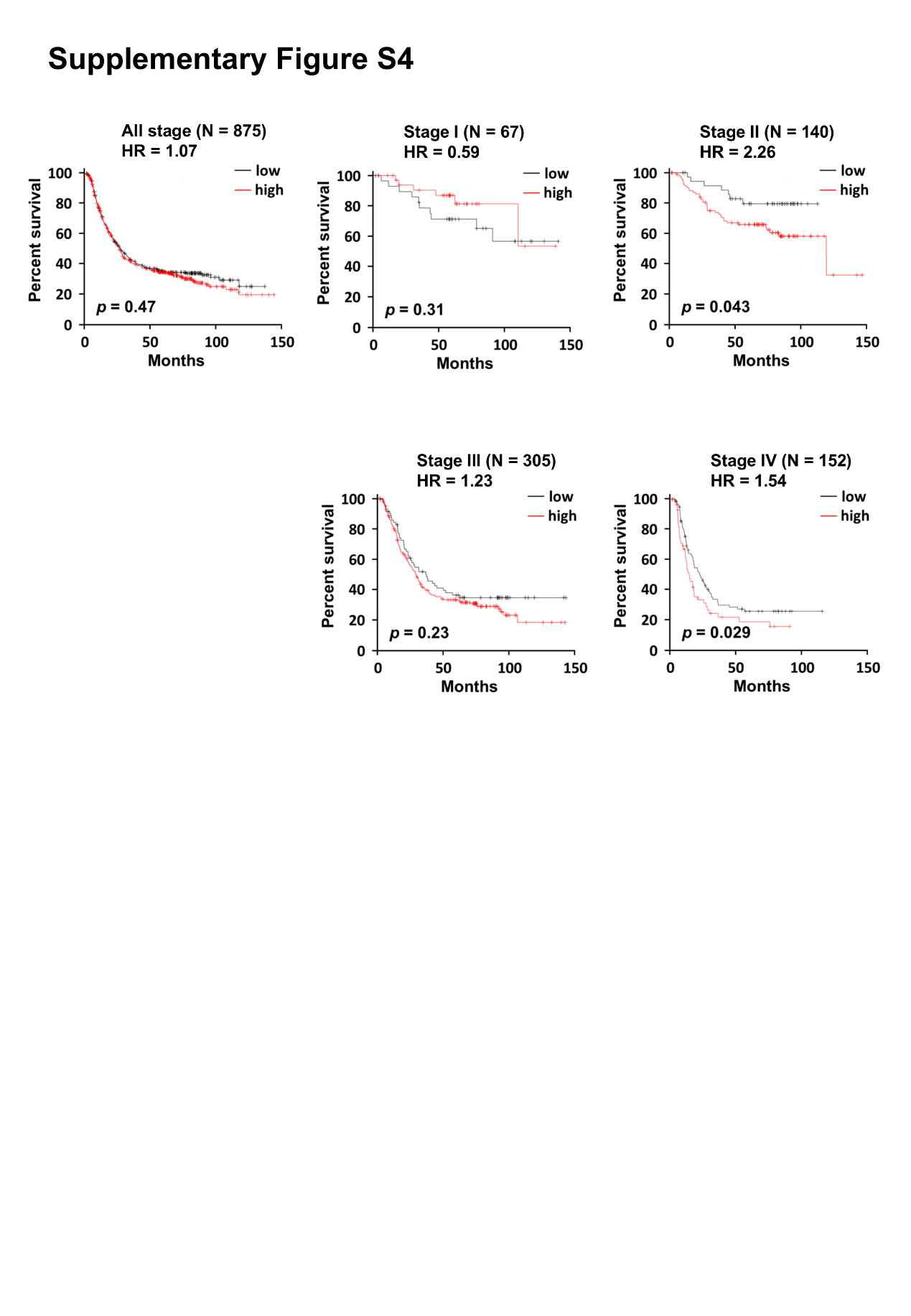


**Supplementary Figure S4. The online database analyses of A20 mRNA expression and survival rates of GC patients.** Kaplan-Meier (KM) Plotter showed that high A20 mRNA expression was strongly associated with lower overall survival rates in stage II (*p* = 0.043) and IV (*p* = 0.029) GC patients.

**
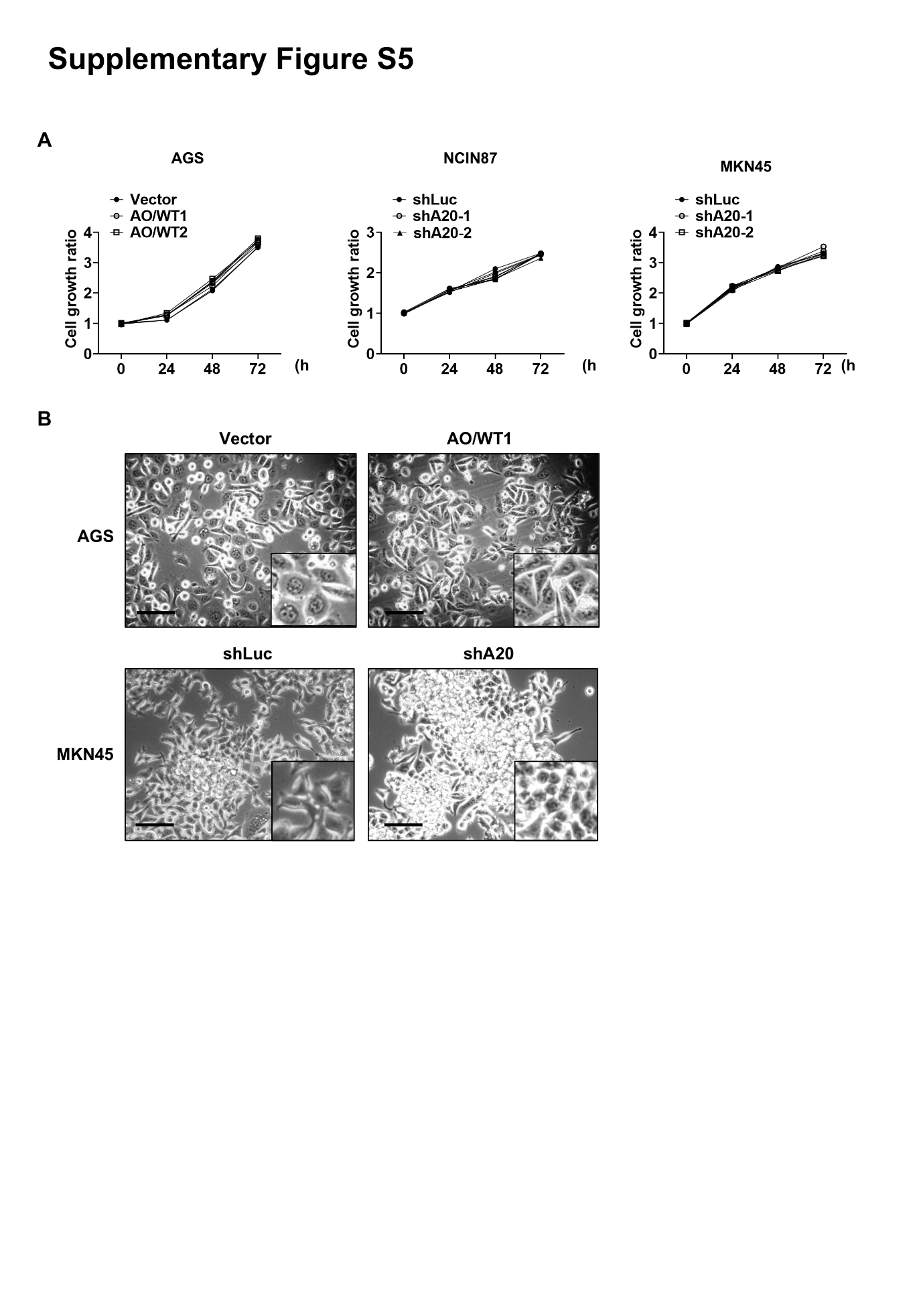
**

**Supplementary Figure S5. A20 expression alters cell morphology but not proliferation in GC cells. A.** Cell proliferation of AGS, NCIN87, MKN45, and their corresponding A20 overexpressing or A20 knockdown derivatives was measured using the MTT assay at 24, 48, and 72 hours. **B.** Cell morphology was assessed by bright-field microscopy in AGS, MKN45, and their A20-manipulated counterparts. Representative images show morphological differences. The scale bar = 50 μm.


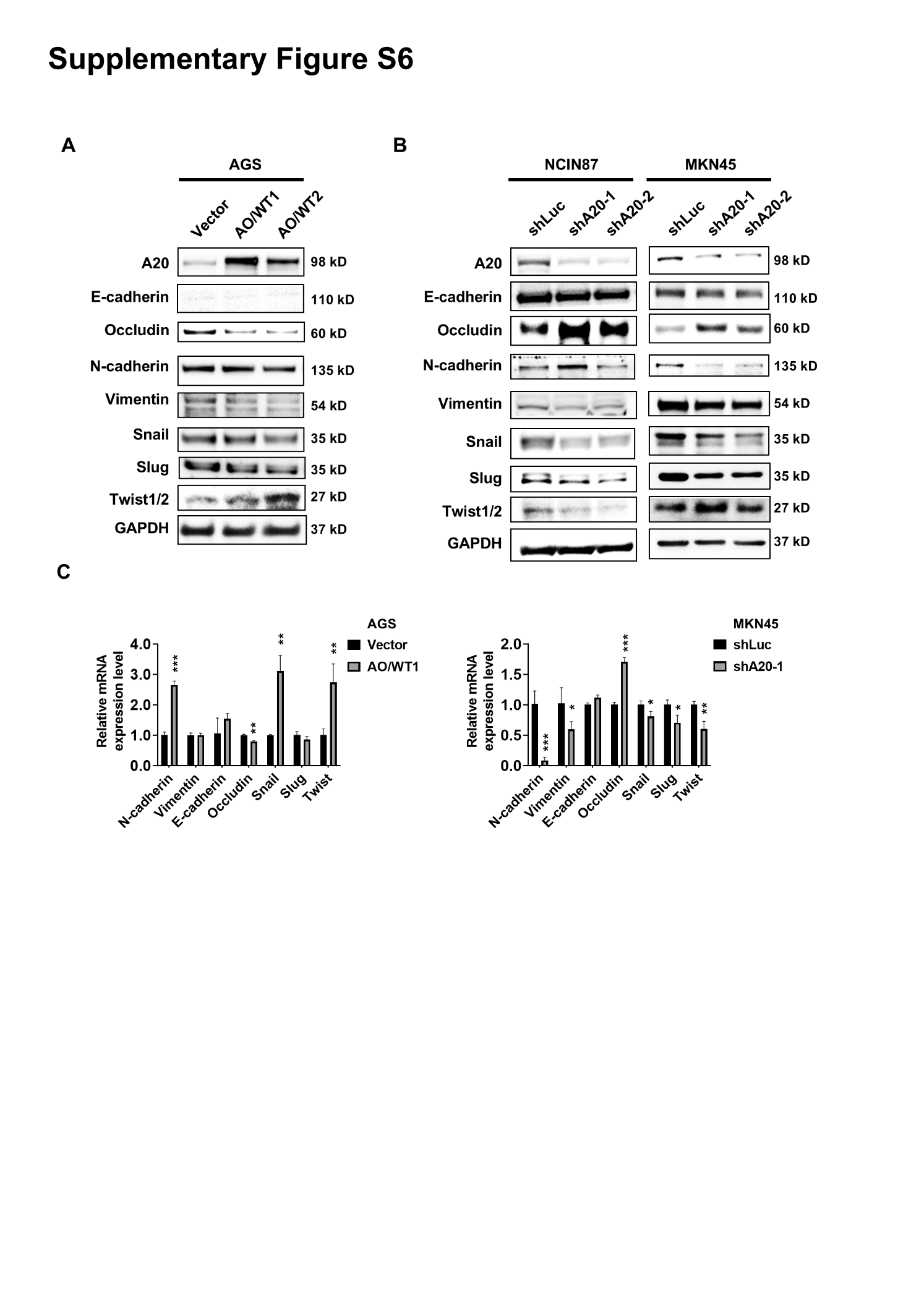


**Supplementary Figure S6. A20 expression is associated with EMT in AGS and MKN45 cells. A and B.** Immunoblot analysis of A20 and EMT-related protein markers in AGS, NCIN87, MKN45 cells, and their A20-overexpressing or A20-knockdown derivatives. **C.** Quantitative RT-PCR analysis of the epithelial markers (E-cadherin and occludin) and the mesenchymal markers (N-cadherin, vimentin, Snail, Slug, and Twist) was performed in AGS, MKN45, and their A20-manipulated derivatives. Bar graphs show relative mRNA expression levels normalized to internal controls. **P* < 0.05; ***P* < 0.01; ****P* < 0.001 versus their corresponding control cells.


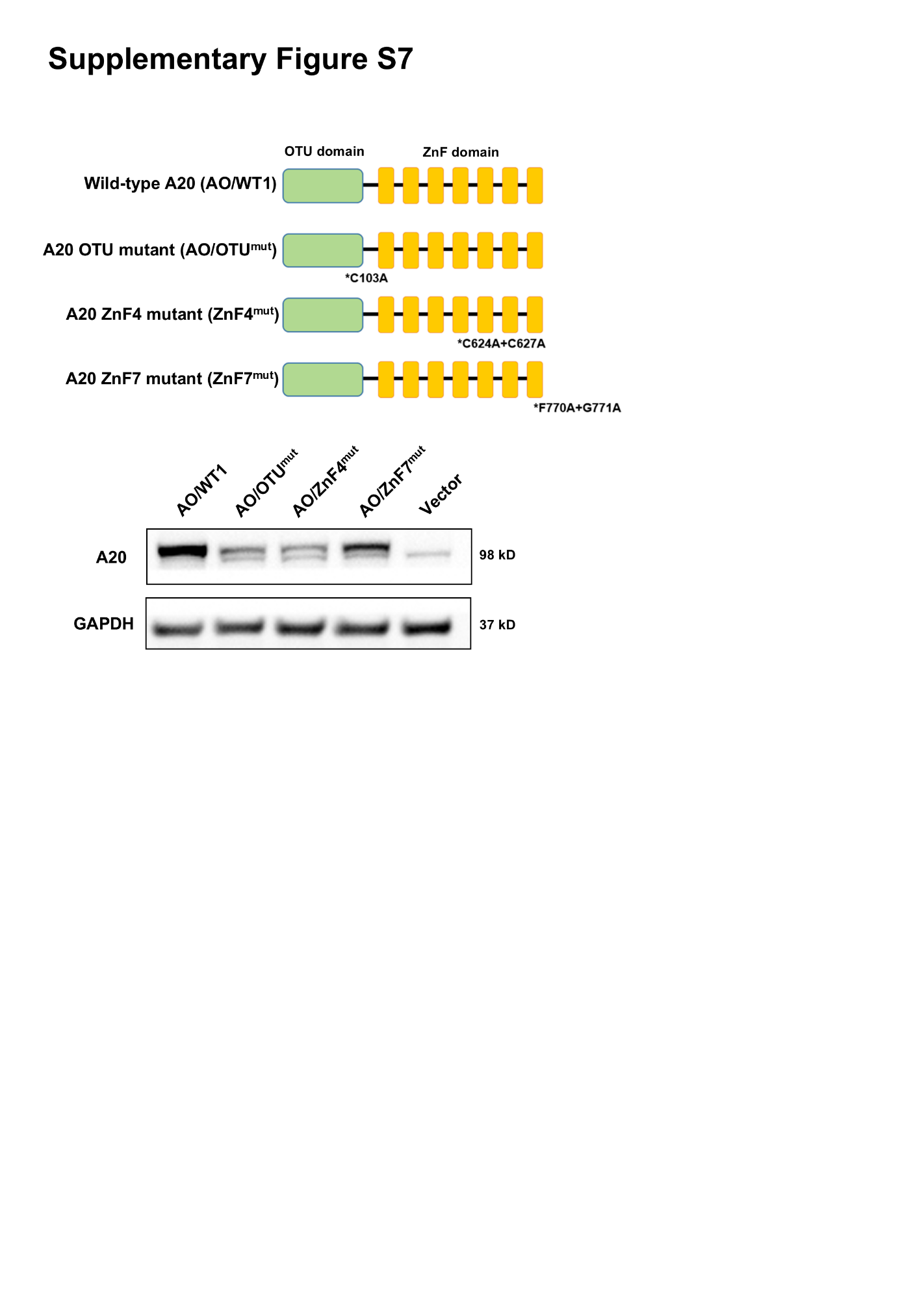


**Supplementary Figure S7.** **Constructs of A20 domain mutants and generation of stable AGS cell lines.** Plasmids encoding wild-type A20 (AO/WT1) and domain-specific A20 mutants, including OTU domain mutant (OTU^mut^), ZnF4 mutant (ZnF4^mut^), and ZnF7 mutant (ZnF7^mut^) were transfected into AGS cells. Stable cell lines expressing wild-type or mutant forms of A20 were established for subsequent functional analyses.


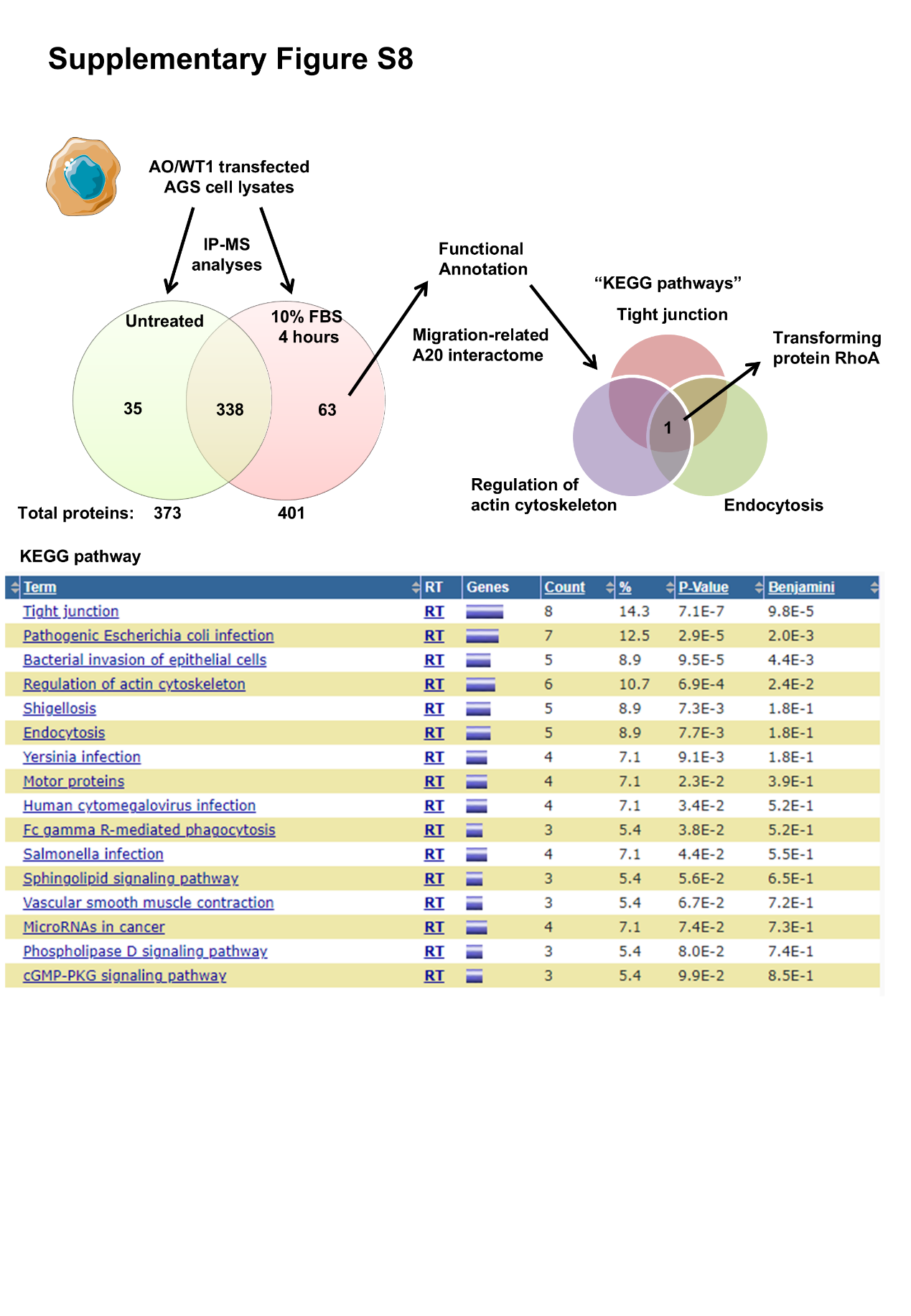


**Supplementary Figure S8. RhoA is the most likely A20-interacting protein involved in occludin degradation.** IP-MS analysis identified 63 A20-interacting proteins in AO/WT1 AGS cells stimulated with FBS for 4 hours. Functional annotation using the KEGG pathway database via DAVID analysis revealed that RhoA is the only A20-interacting protein simultaneously involved in tight junction regulation, endocytosis, and actin cytoskeleton dynamics, suggesting its central role in A20-mediated occludin degradation.

**
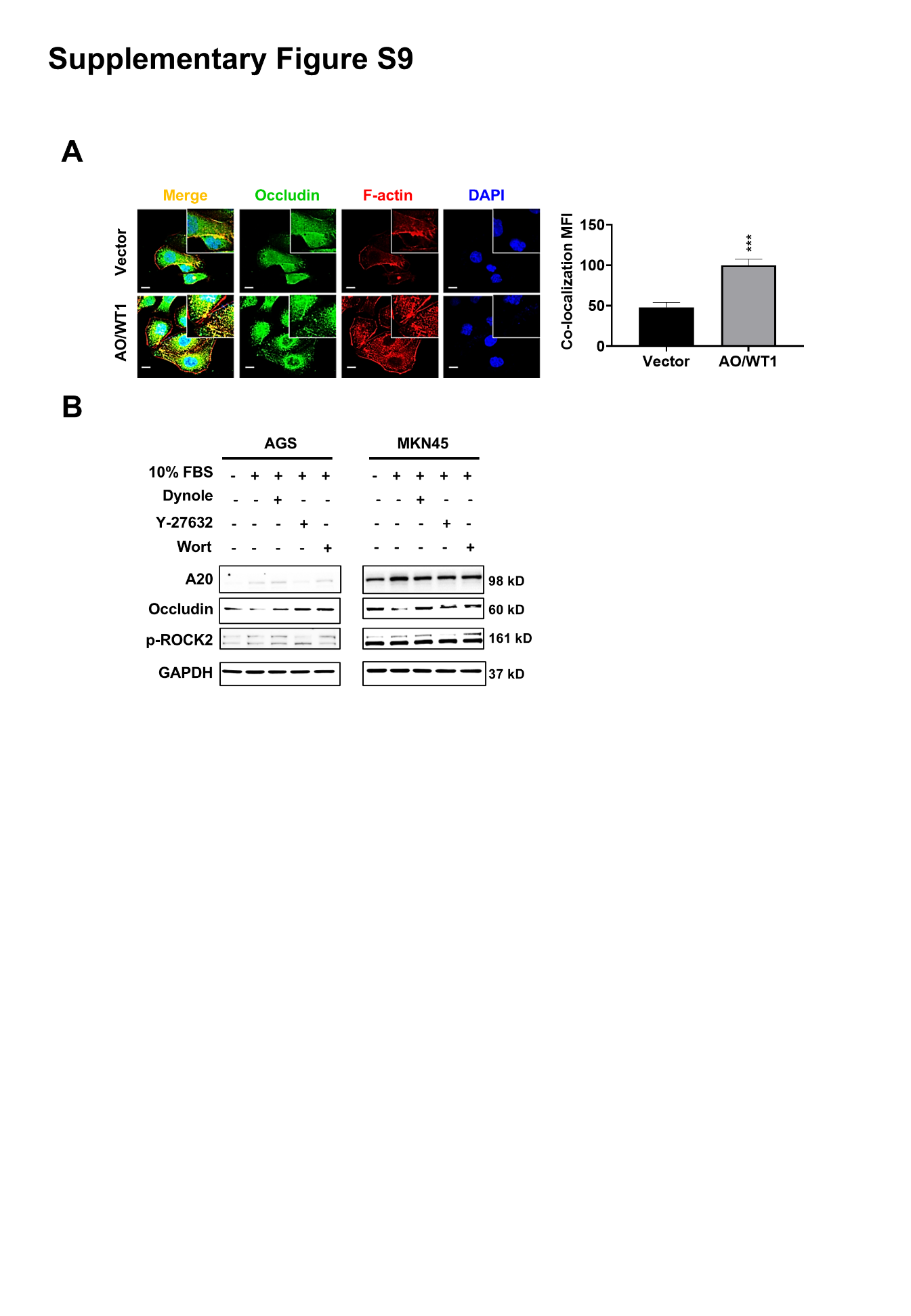
**

**Supplementary Figure S9. Inhibition of endocytosis or ROCK2 phosphorylation prevents occludin degradation. A.** Confocal IF staining showed increased cytosolic co-localization of occludin (green) and F-actin (red) in AO/WT1 AGS cells. Scale bar = 10 μm. **B.** Pharmacological inhibition of endocytosis with Dynole (10 μM, a dynamin inhibitor) or wortmannin (Wort, 100 nM), and inhibition of ROCK2 activity with Y-27632 (20 μM), effectively blocked FBS-induced occludin degradation in both AGS and MKN45 cells.


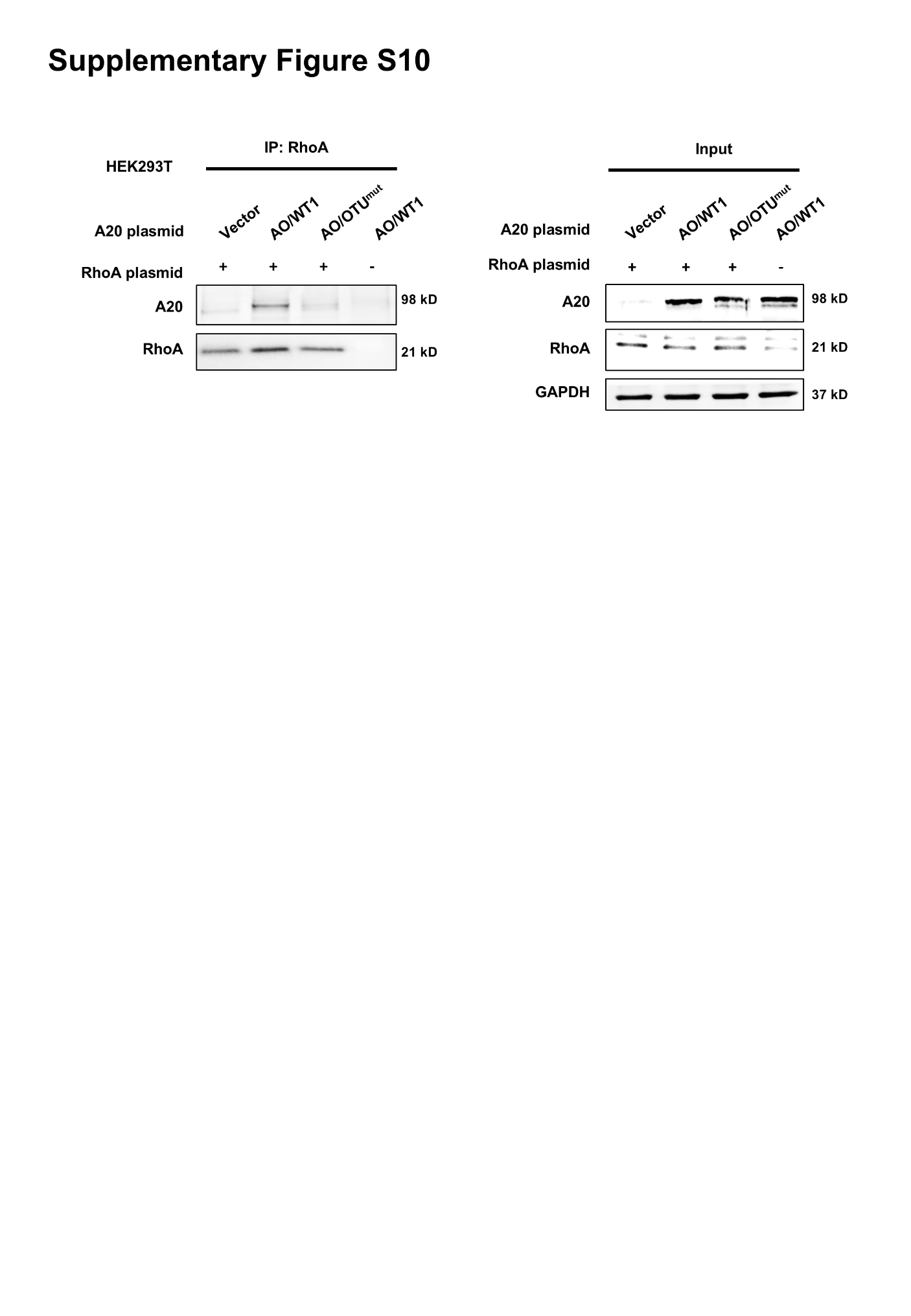


**Supplementary Figure S10. A20 interacts with RhoA in HEK293T cells.** Co-IP was performed in HEK293T cells co-transfected with A20 and RhoA. The interaction between A20 and RhoA was confirmed and found to be disrupted by mutation in the A20 OTU domain.


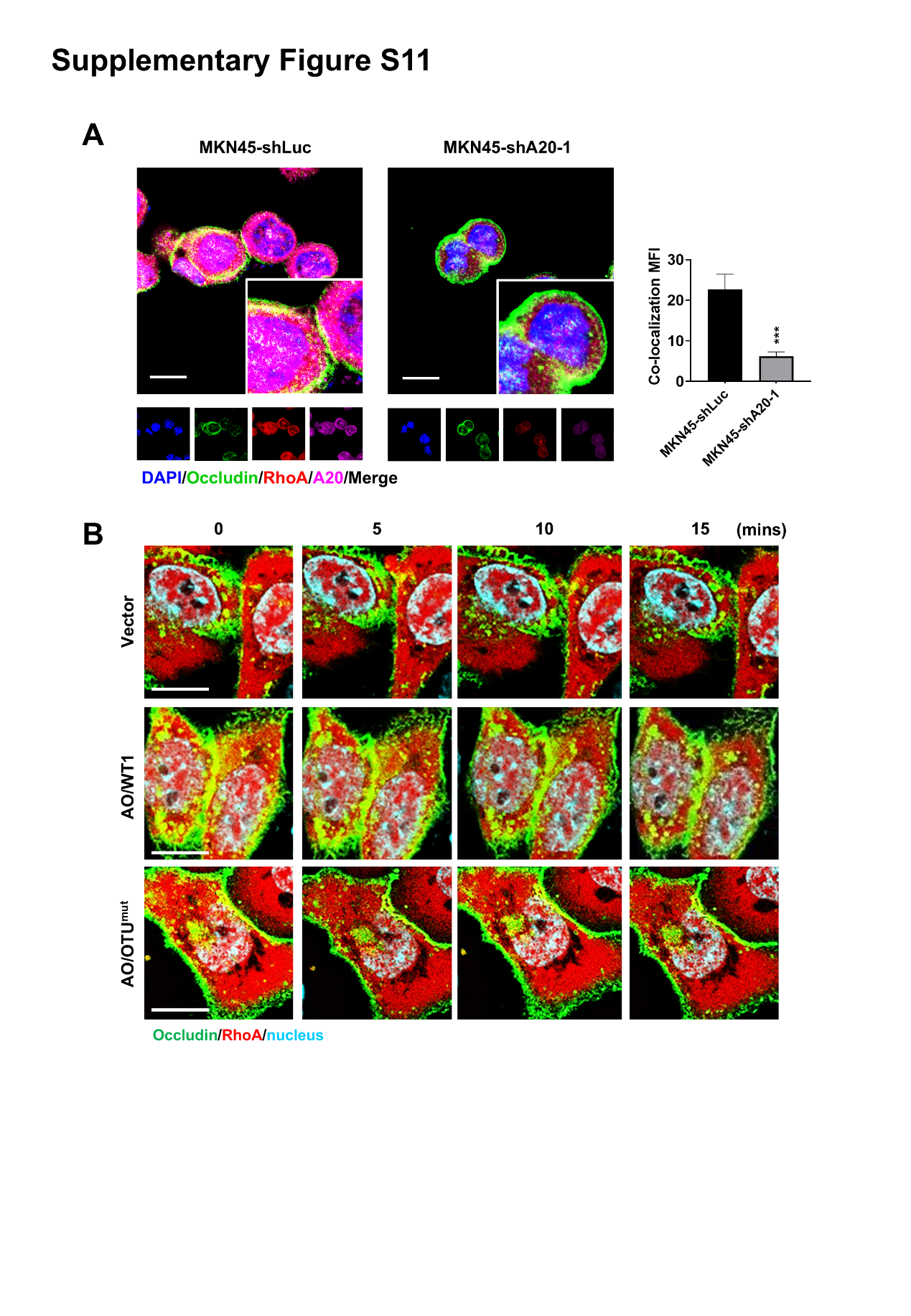


**Supplementary Figure S11. A20 co-localizes with RhoA and occludin. A.** Triple IF staining for A20, RhoA, and endocytic occludin was performed in MKN45-shLuc and MKN45-shA20-1 cells. A20 knockdown reduced the co-localization of all three proteins (****P* < 0.001). Scale bar = 10 μm. **B.** Time-lapse confocal imaging showed enhanced cytosolic co-localization and dynamic movement of occludin and RhoA in AO/WT1 AGS cells compared to vector control or OTU^mut^ AGS cells over a 15-minute period. Scale bar = 10 μm.

**Supplementary Table S1. Primers for A20 mutagenesis**

| **Mutation site** | **Forward (5’-3’)** | **Reverse (5’-3’)** |
| --- | --- | --- |
| C103A | AACGGTGACGGCAATGCCCTCATGCATGCCACT | AGTGGCATGCATGAGGGCATTGCCGTCACCGTT |
| C624A/C627A | TTTTCTCTGTACTCGATGAAAGCCAGTGTGGCAAAGCCCTTGTTTTCTGGAGTC | GACTCCAGAAAACAAGGGCTTTGCCACACTGGCTTTCATCGAGTACAGAGAAAA |
| F770A/G771A | CGTTGCACTTGGCATTGGCAGCATGATCACAGGCGGGGG | CCCCCGCCTGTGATCATGCTGCCAATGCCAAGTGCAACG |

**Supplementary Table S2. Primers for Sanger sequencing of A20**

| **Clone name** | **Forward (5’-3’)** | **Reverse (5’-3’)** |
| --- | --- | --- |
| C103A | GCCCTCATCGACAGAAACAT | GAACAGCGCCTTCCTCAGTA |
| C624A/C627A | GCTGCGTGTATTTTGGGACT | GTGGGACTGACTTTCCCTGA |
| F770A/G771A | CTGCATGGAGTGTCAGCATC | AAGCATTCGTTGCAGTAGCC |

**Supplementary Table S3. Primers for real-time PCR**

| **Gene name** | **Forward (5’-3’)** | **Reverse (5’-3’)** |
| --- | --- | --- |
| A20 | GAGAGCACAATGGCTGAACA | TCCAGTGTGTATCGGTGCAT |
| N-cadherin | CATCATCATCCTGCTTATCCTTGT | GGTCTTCTTCTCCTCCACCTTCT |
| E-cadherin | TGCCCAGAAAATGAAAAAGG | GTGTATGTGGCAATGCGTTC |
| Vimentin | TACAGGAAGCTGCTGGAAGG | ACCAGAGGGAGTGAATCCAG |
| Occludin | CCACGCCGGTTCCTGAAGTGG | TCACAGGACTCGCCGCCAGT |
| Snail | AAGGATCTCCAGGCTCGAAAG | GCTTCGGATGTGCATCTTGA |
| Slug | TGGTTGCTTCAAGGACACAT | GTTGCAGTGAGGGCAAGAA |
| Twist1/2 | AAGGCATCACTATGGACTTTCTCT | GCCAGTTTGATCCCAGTATTTT |
| GAPDH | AATCCCATCACCATCTTCCA | TGGACTCCACGACGTACTCA |

**Supplementary Table S4. List of antibodies**

| A20 | GTX62211 | WB = 1:1000 |
| --- | --- | --- |
| N-cadherin | GTX127345 | WB = 1:1000 |
| E-cadherin | GTX100443 | WB = 1:1000 |
| Vimentin | GTX100619 | WB = 1:1000 |
| Occludin | GTX114949 | WB = 1:1000; IF = 1:200 |
| Snail | GTX125918 | WB = 1:1000 |
| Slug | GTX128796 | WB = 1:1000 |
| Twist1/2 | GTX127310 | WB = 1:1000 |
| RhoA | GTX01410 | WB = 1:1000 |
| GAPDH | GTX627408 | WB = 1:5000 |
| ROCK2 | GTX102619 | WB = 1:1000 |
| p-ROCK2 (S1366) | GTX636103 | WB = 1:1000 |
| ERK1/2 | GTX134462 | WB = 1:2000 |
| p-ERK1/2 (T202/Y204/T185/Y187) | GTX635617 | WB = 1:2000 |
| DDK | 14793S | IP = 1 μg; PLA = 1:200 |
| A20 | GTX11900 | IP = 1 μg; IF=1:200 |
| RhoA | sc-418 | IP = 1 μg; IF = 1:100; PLA = 1:100 |
| Occludin | 33-1500 | IF = 1:200 |
| Goat Anti-Rabbit IgG (HRP) | GTX213110-01 | WB = 1:5000 |
| Goat Anti-Mouse IgG (HRP) | GTX213111-01 | WB = 1:5000 |
| Goat Anti-Rabbit IgG (DyLight488) | GTX213110-04 | IF = 1:1000 |
| Goat Anti-Rabbit IgG (DyLight594) | GTX213111-05 | IF = 1:1000 |
| Goat Anti-Mouse IgG (DyLight633) | GTX76787 | IF = 1:1000 |

**Supplementary Table S5. Demographic characteristics of GC patients in the full analysis set**

| **Characteristic** | **Total (n=122)** | **Percentage (%)** |
| --- | --- | --- |
| **Age median=68.0 (24 - 92)** |  |  |
| <65 | 49 | 40.2 |
| ≥65 | 73 | 59.8 |
| **Follow-up time median= 40.95 month** | | |
| **Gender** |  |  |
| male | 74 | 60.7 |
| Female | 48 | 39.3 |
| **Subserosal invasion** |  |  |
| negative | 71 | 58.2 |
| positive | 51 | 41.8 |
| **Lymph node metastasis** |  |  |
| negative | 55 | 45.1 |
| positive | 67 | 54.9 |
| **Stage** |  |  |
| I, II, and III | 95 | 77.9 |
| IV | 27 | 22.1 |
| **Perineural invasion** |  |  |
| negative | 57 | 46.7 |
| positive | 65 | 53.3 |
| **Tumor emboli** |  |  |
| negative | 31 | 25.4 |
| positive | 91 | 74.6 |
| **Lauren classification** |  |  |
| Intestinal | 53 | 43.4 |
| Diffuse + mix | 69 | 56.6 |
| **A20 expression** |  |  |
| Grade 1 | 11 | 9.0 |
| Grade 2 | 30 | 24.6 |
| Grade 3 | 36 | 29.5 |
| Grade 4 | 45 | 36.9 |
| Intestinal: intestinal type gastric cancer; Diffuse + mix: diffuse type and mix type gastric cancer; values in boldface indicate *p* < 0.05. | | |

**Supplementary Table S6. Correlation between A20 expression and clinicopathological profiles**

| **Characteristic** | Low A20 expression | High A20 expression | ***p*-value** |
| --- | --- | --- | --- |
| **Age** |  |  |  |
| <65 | 18 (43.90) | 31 (38.27) | 0.564 |
| ≥65 | 23 (56.10) | 50 (61.73) |  |
| **Gender** |  |  |  |
| male | 19 (46.34) | 55 (67.90) | **0.031** |
| Female | 22 (53.66) | 26 (32.10) |  |
| **Subserosal invasion** |  |  |  |
| negative | 31 (75.61) | 40 (49.38) | **0.007** |
| positive | 10 (24.39) | 41 (50.62) |  |
| **Lymph node metastasis** |  |  |  |
| negative | 29 (70.73) | 26 (32.10) | **<0.001** |
| positive | 12 (29.27) | 55 (67.90) |  |
| **Stage** |  |  |  |
| I, II, and III | 38 (92.68) | 57 (70.37) | **0.005** |
| IV | 3 (7.32) | 24 (29.63) |  |
| **Perineural invasion** |  |  |  |
| negative | 29 (70.73) | 28 (34.57) | **<0.001** |
| positive | 12 (29.27) | 53 (65.43) |  |
| **Tumor emboli** |  |  |  |
| negative | 20 (48.78) | 11 (13.58) | **<0.001** |
| positive | 21 (51.22) | 70 (86.42) |  |
| **Lauren classification** |  |  |  |
| Intestinal | 21 (51.22) | 32 (39.51) | 0.249 |
| Diffuse + mix | 20 (48.78) | 49 (60.49) |  |
| A20 low expression: grade 1 and grade 2; A20 high expression: grade 3 and grade 4; Intestinal: intestinal type gastric cancer; Diffuse + mix: diffuse type and mix type gastric cancer; values in boldface indicate *p* < 0.05. | | | |

**Supplementary Table S7. Univariate and multivariate analyses of survival in 122 GC patients**

| **Factor** | **Progression-free survival** | | | | **Overall survival** | | | |
| --- | --- | --- | --- | --- | --- | --- | --- | --- |
|  | **Univariate** | | **Multivariate** | | **Univariate** | | **Multivariate** | |
|  | **HR**  **(95% CI)** | ***p*-value** | **HR**  **(95% CI)** | ***p-*value** | **HR**  **(95% CI)** | ***p*-value** | **HR**  **(95% CI)** | ***p*-value** |
| Age > 65 | 1.564  (0.897-2.725) | 0.115 | - | **-** | 1.576  (0.904-2.745) | 0.108 | **1.774**  **(1.015-3.098)** | **0.044** |
| Gender female | 0.800  (0.467-1.372) | 0.417 | **-** | **-** | 0.827  (0.482-1.416) | 0.488 | **-** | **-** |
| Subserosa (+) | **2.296**  **(1.358-3.880)** | **0.002** | **-** | **-** | **2.193**  **(1.298-3.705)** | **0.003** | **-** | **-** |
| LNM (+) | **3.663**  **(1.999-6.714)** | **<0.001** | **-** | **-** | **3.551**  **(1.938-6.506)** | **<0.001** | **-** | **-** |
| Stage IV | **7.418**  **(4.271-12.884)** | **<0.001** | **5.465**  **(3.107-9.612)** | **<0.001** | **7.342**  **(4.236-12.726)** | **<0.001** | **5.658**  **(3.202-9.999)** | **<0.001** |
| PNI (+) | **2.453**  **(1.410-4.266)** | **0.001** | **-** | **-** | **2.476**  **(1.424-4.307)** | **0.001** | **-** | **-** |
| Tumor emboli (+) | **2.108**  **(1.063-4.180)** | **0.033** | **-** | **-** | **2.194**  **(1.106-4.352)** | **0.025** | **-** | **-** |
| Diffuse + mix | **2.262**  **(1.291-3.963)** | **0.004** | **-** | **-** | **2.203**  **(1.258-3.858)** | **0.006** | **-** | **-** |
| A20 + | **4.980**  **(2.407-10.301)** | **<0.001** | **3.530**  **(1.684-7.400)** | **0.001** | **4.912**  **(2.382-10.132)** | **<0.001** | **3.396**  **(1.608-7.169)** | **0.001** |
