# Supplementary material for: A20 enhances the migration and metastasis of gastric cancer cells by promoting occludin degradation
Source: Cell Death Discov. 2026 Mar 28;12:206. doi: 10.1038/s41420-026-03082-2 (PMC13153315; doi:10.1038/s41420-026-03082-2)

Figure 1B

A20

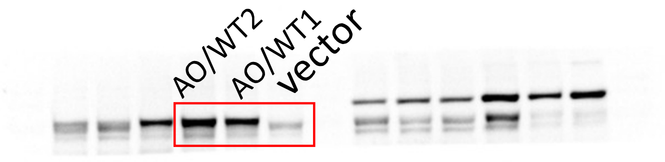

GAPDH

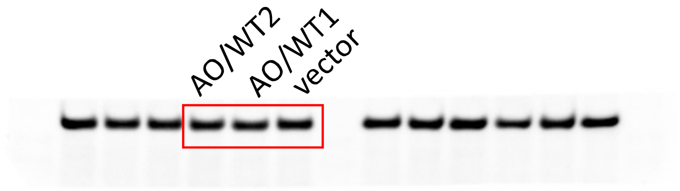

Figure 3C

Occludin

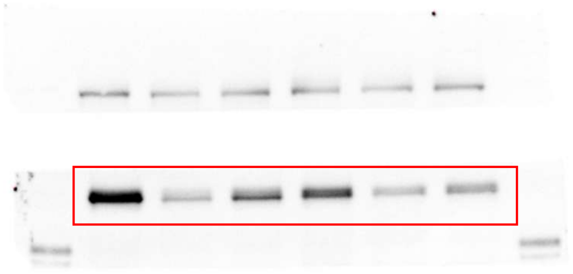

GAPDH

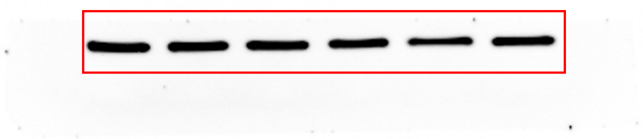

Figure 4B

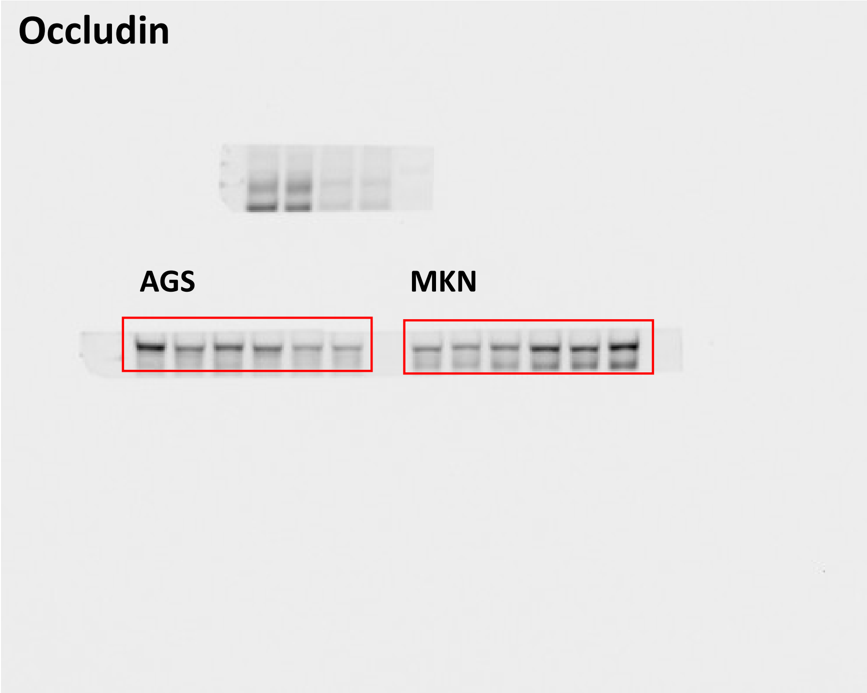

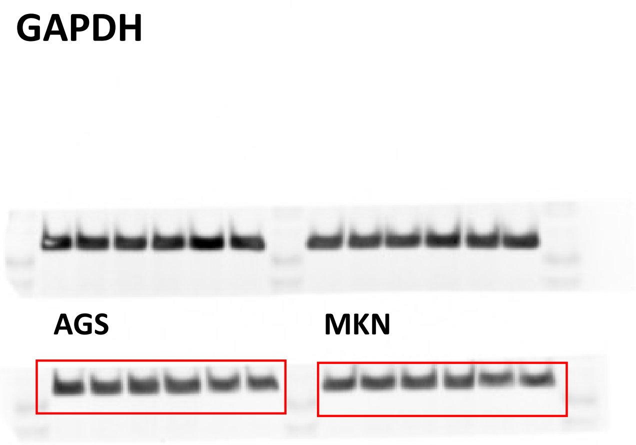

A20

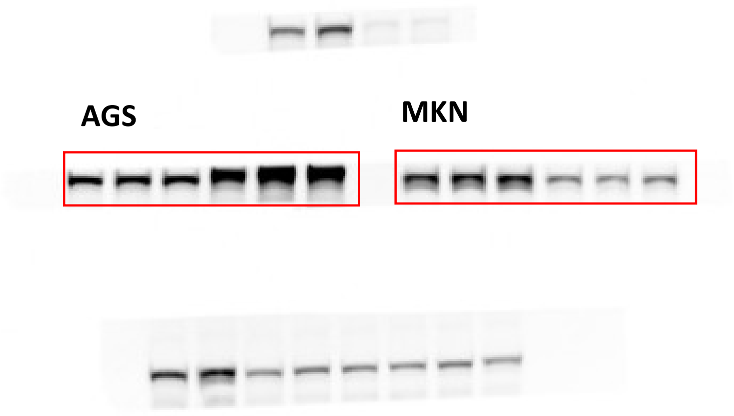

Figure 6D

A20

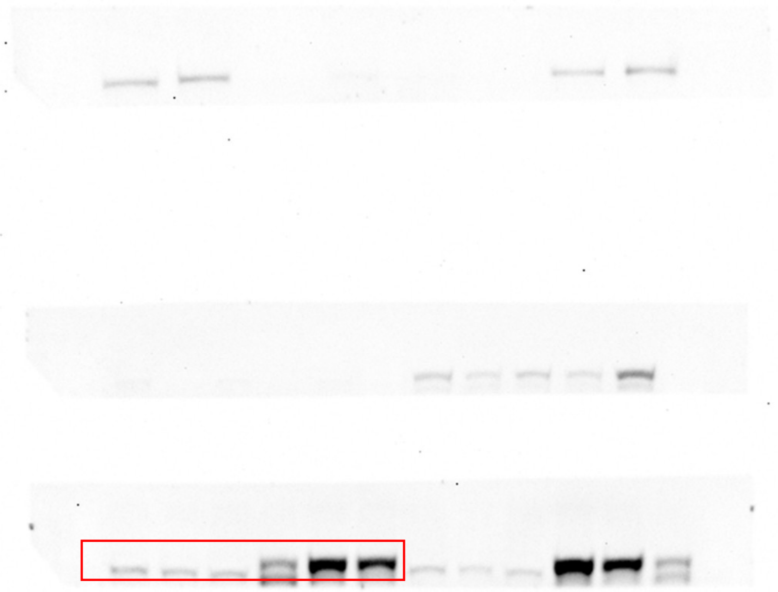

Occludin

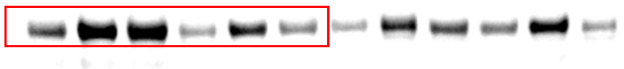

RhoA

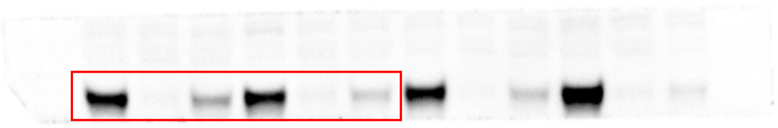

GAPDH

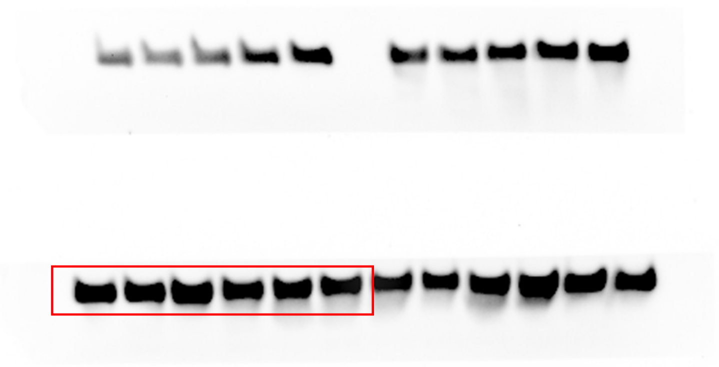

Figure 6F

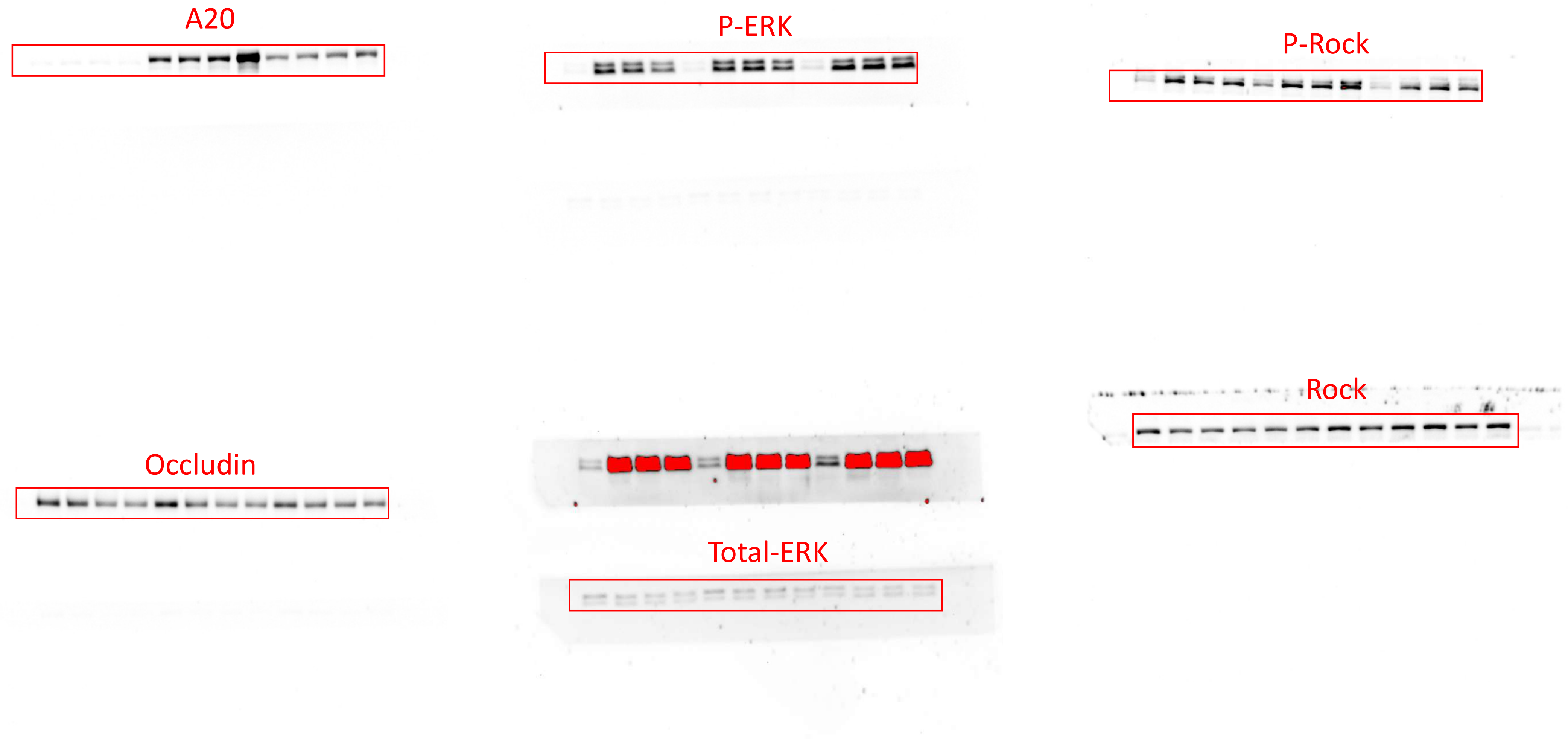

IP:A20

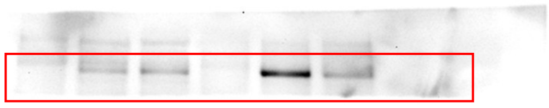

IP: RhoA

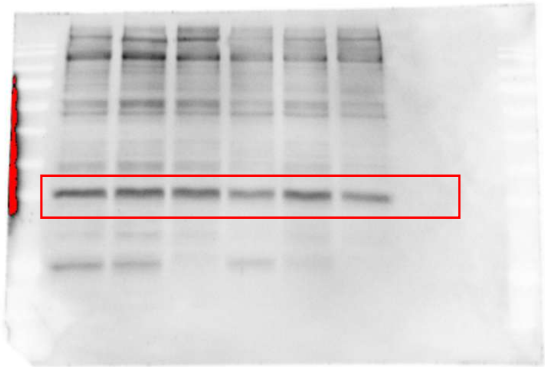

Inpute: A20

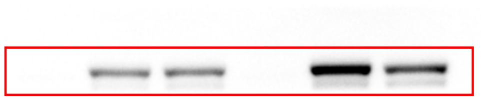

Inpute: RhoA

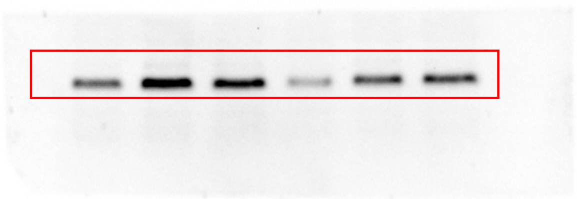

Inpute: GAPDH

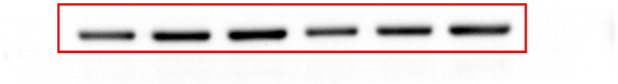

Figure 7G

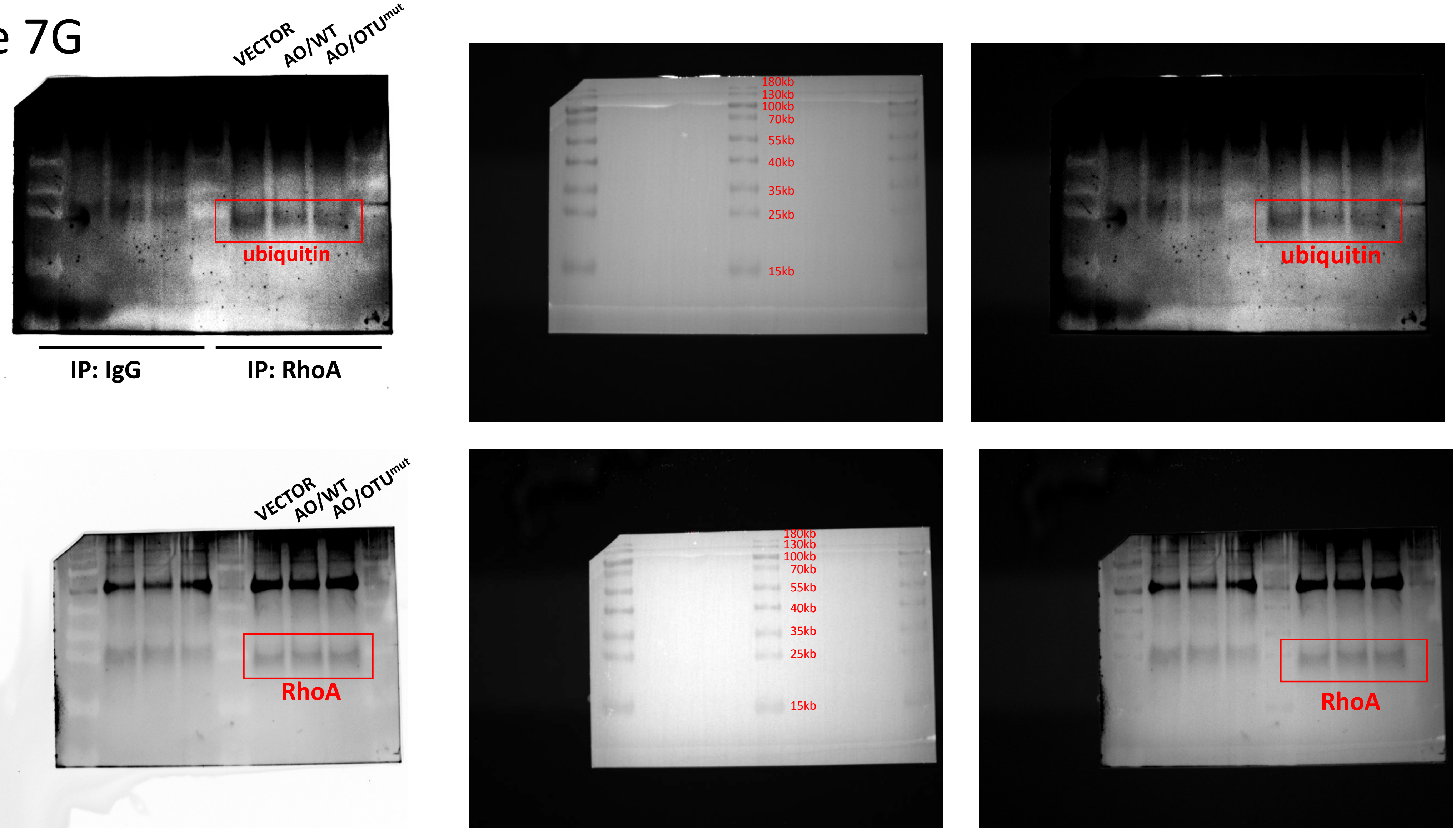

VECTOR
AO/WT
AO/OTUmut
ubiquitin
IP: IgG
IP: RhoA
180kb
130kb
100kb
70kb
55kb
40kb
35kb
25kb
15kb
ubiquitin
VECTOR
AO/WT
AO/OTUmut
RhoA
180kb
130kb
100kb
70kb
55kb
40kb
35kb
25kb
15kb
RhoA

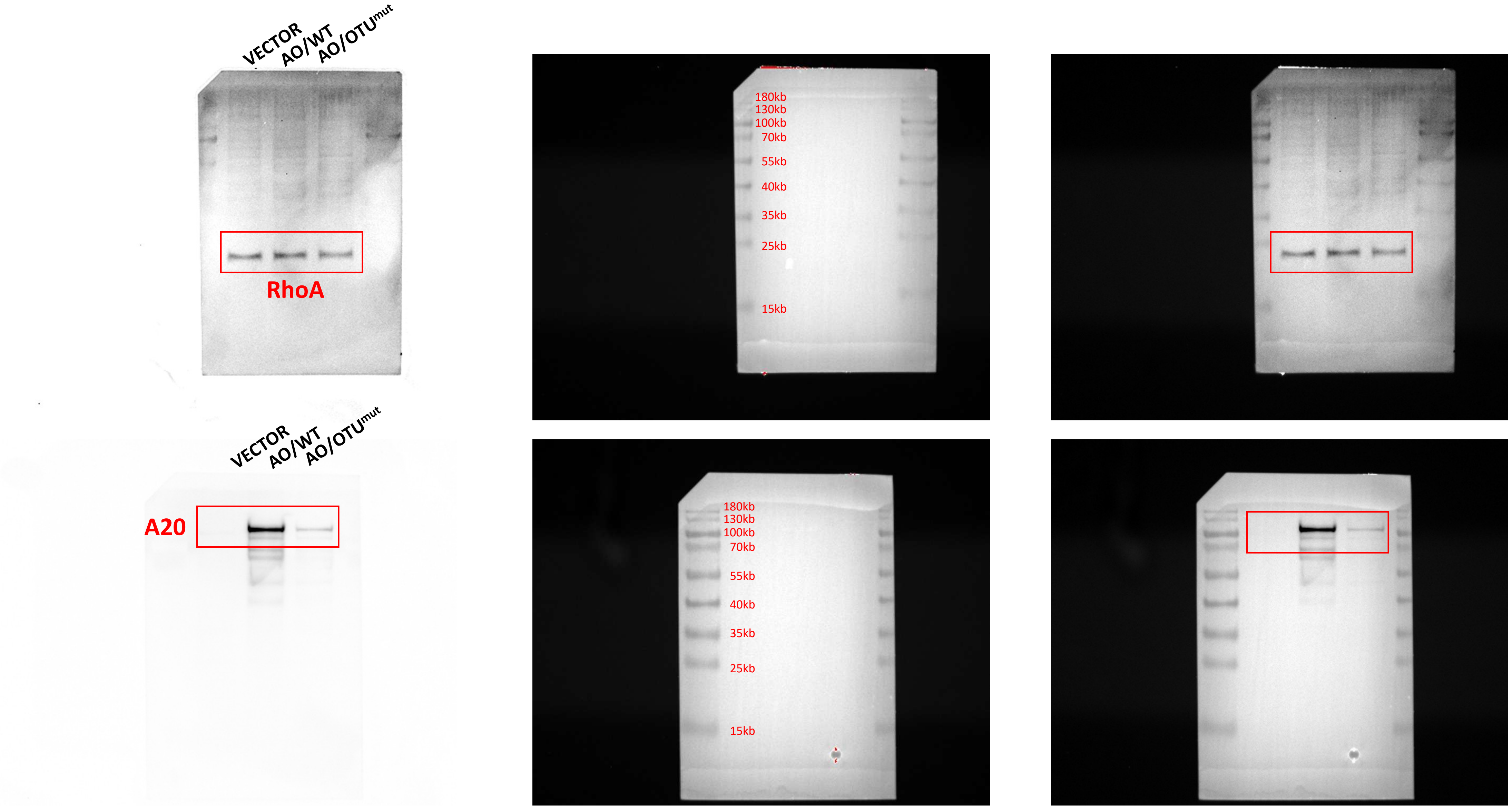
VECTOR
AO/WT
AO/OTUmut
RhoA
180kb
130kb
100kb
70kb
55kb
40kb
35kb
25kb
15kb
VECTOR
AO/WT
AO/OTUmut
A20
180kb
130kb
100kb
70kb
55kb
40kb
35kb
25kb
15kb

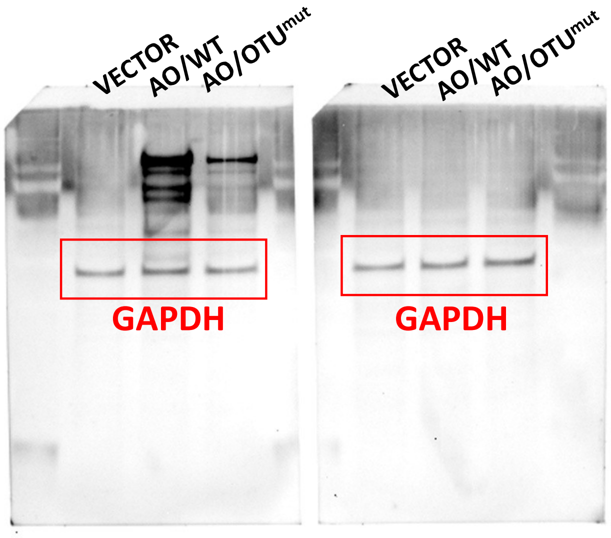
VECTOR
AO/WT
AO/OTUmut
VECTOR
AO/WT
AO/OTUmut
GAPDH
GAPDH

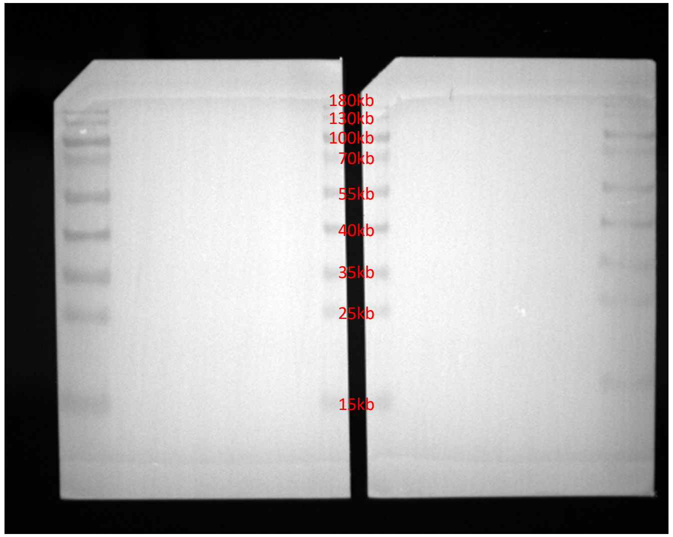
180kb
130kb
100kb
70kb
55kb
40kb
35kb
25kb
15kb

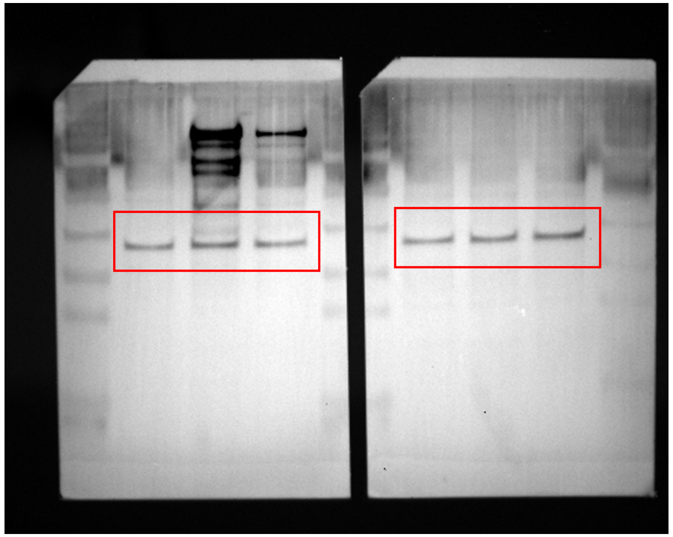

Supplementary Figure S7

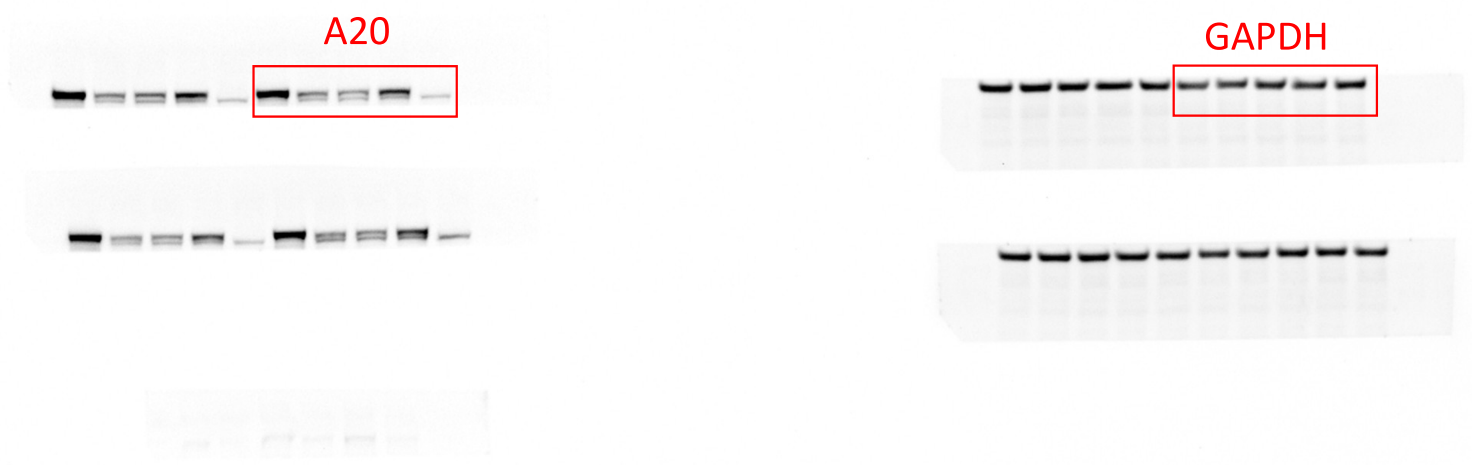

Supplement: Supplementary file 3 — raw data of western figure [file 41420_2026_3082_MOESM3_ESM.pdf]
