# Supplementary material for: A20 enhances the migration and metastasis of gastric cancer cells by promoting occludin degradation
Source: Cell Death Discov. 2026 Mar 28;12:206. doi: 10.1038/s41420-026-03082-2 (PMC13153315; doi:10.1038/s41420-026-03082-2)
Supplement: Supplementary file 4 — Western raw data for some figures [file 41420_2026_3082_MOESM4_ESM.pptx]

## Slide 1
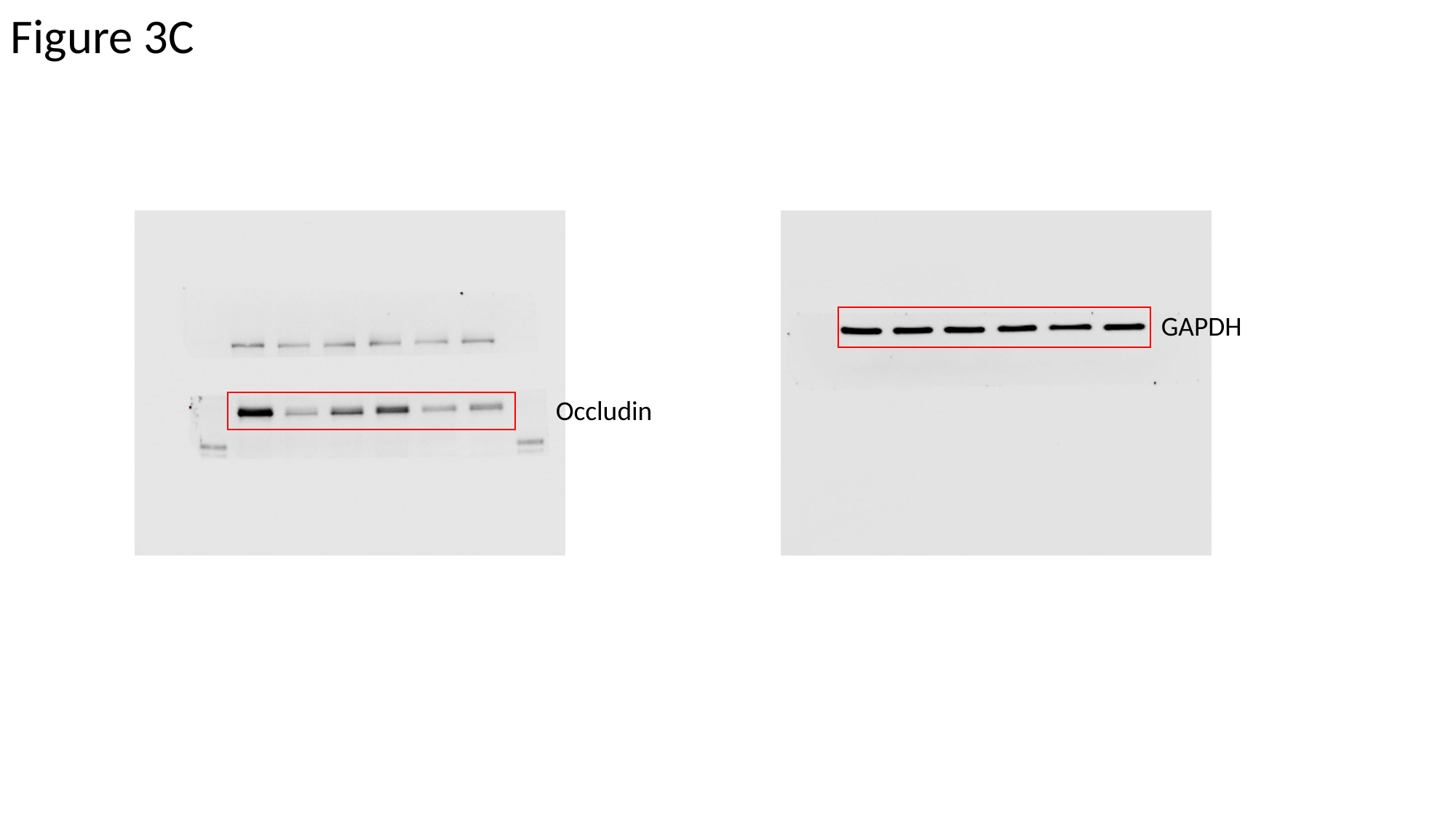

Figure 3C
GAPDH
Occludin

## Slide 2
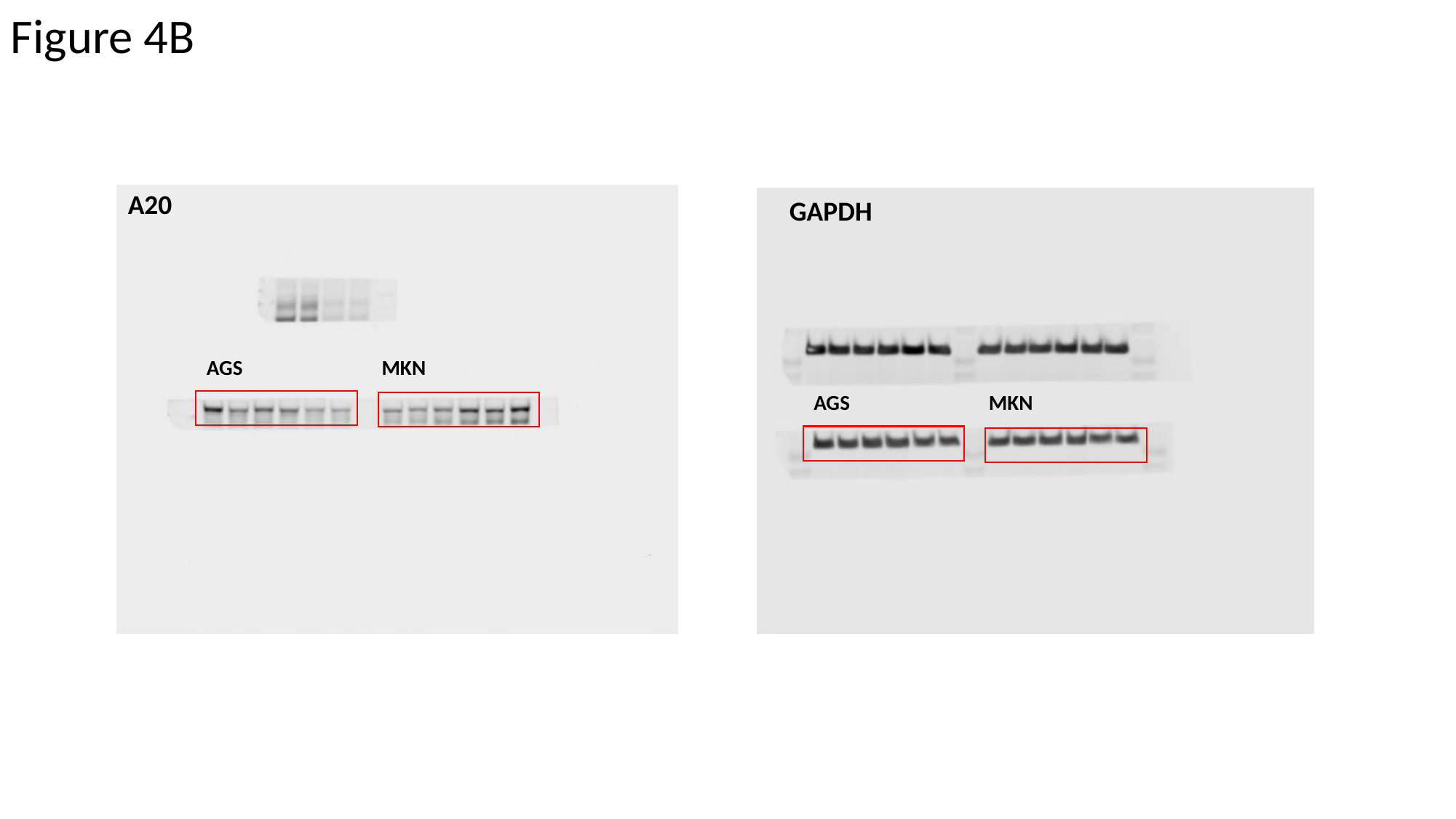

Figure 4B
A20
GAPDH
AGS
MKN
AGS
MKN

## Slide 3
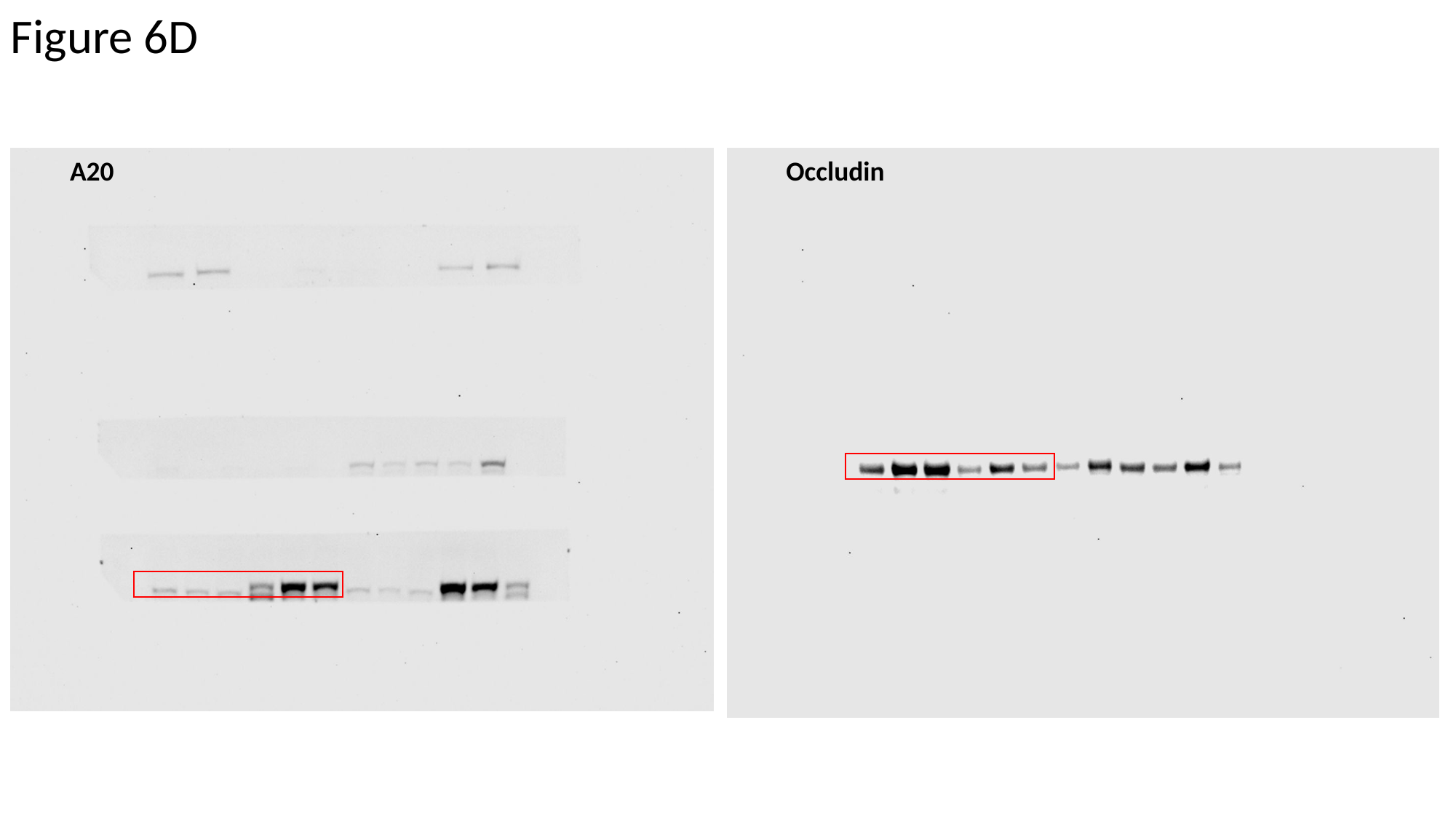

Figure 6D
A20
Occludin

## Slide 4
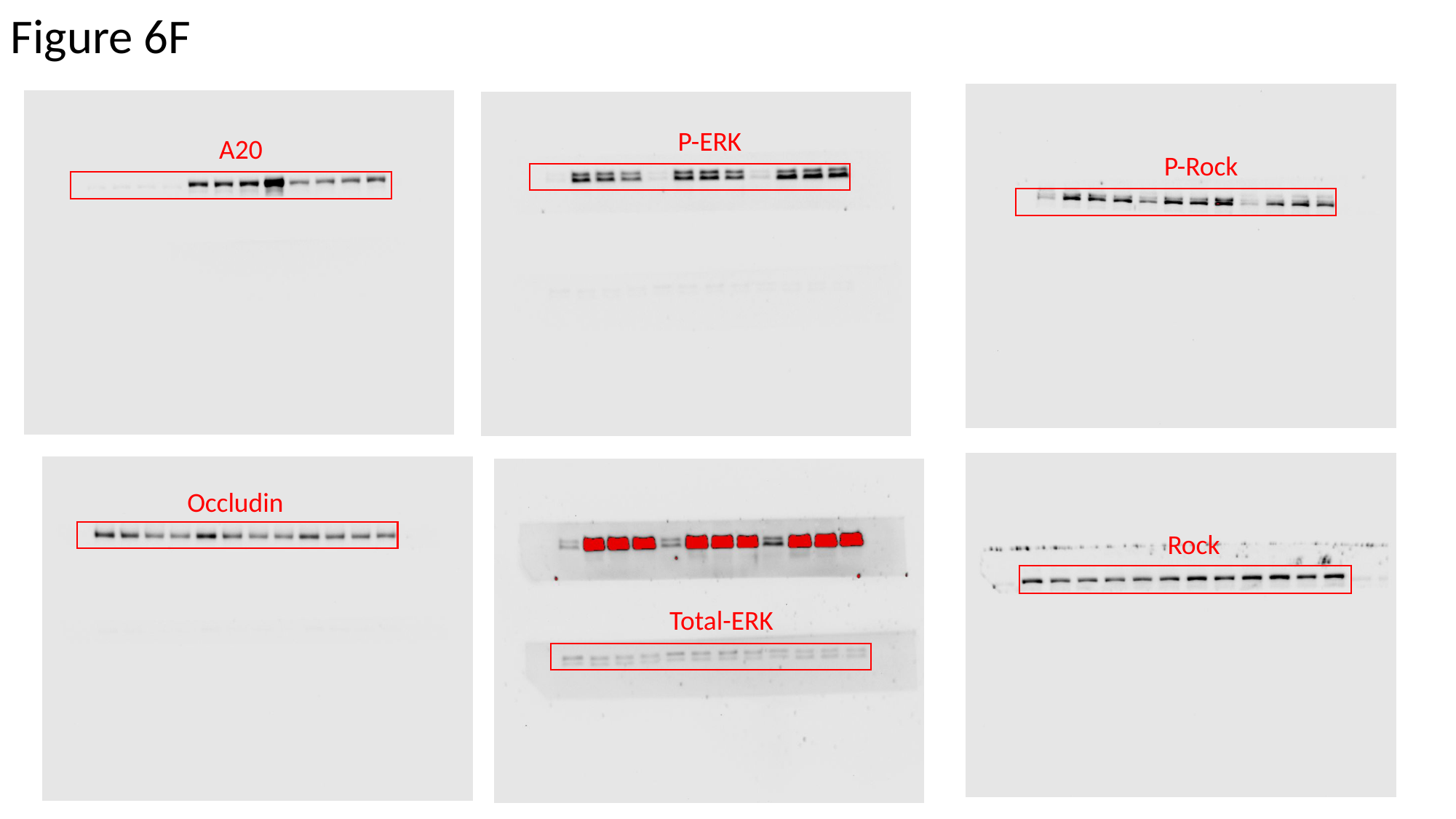

Figure 6F
P-ERK
A20
P-Rock
Occludin
Rock
Total-ERK

## Slide 5
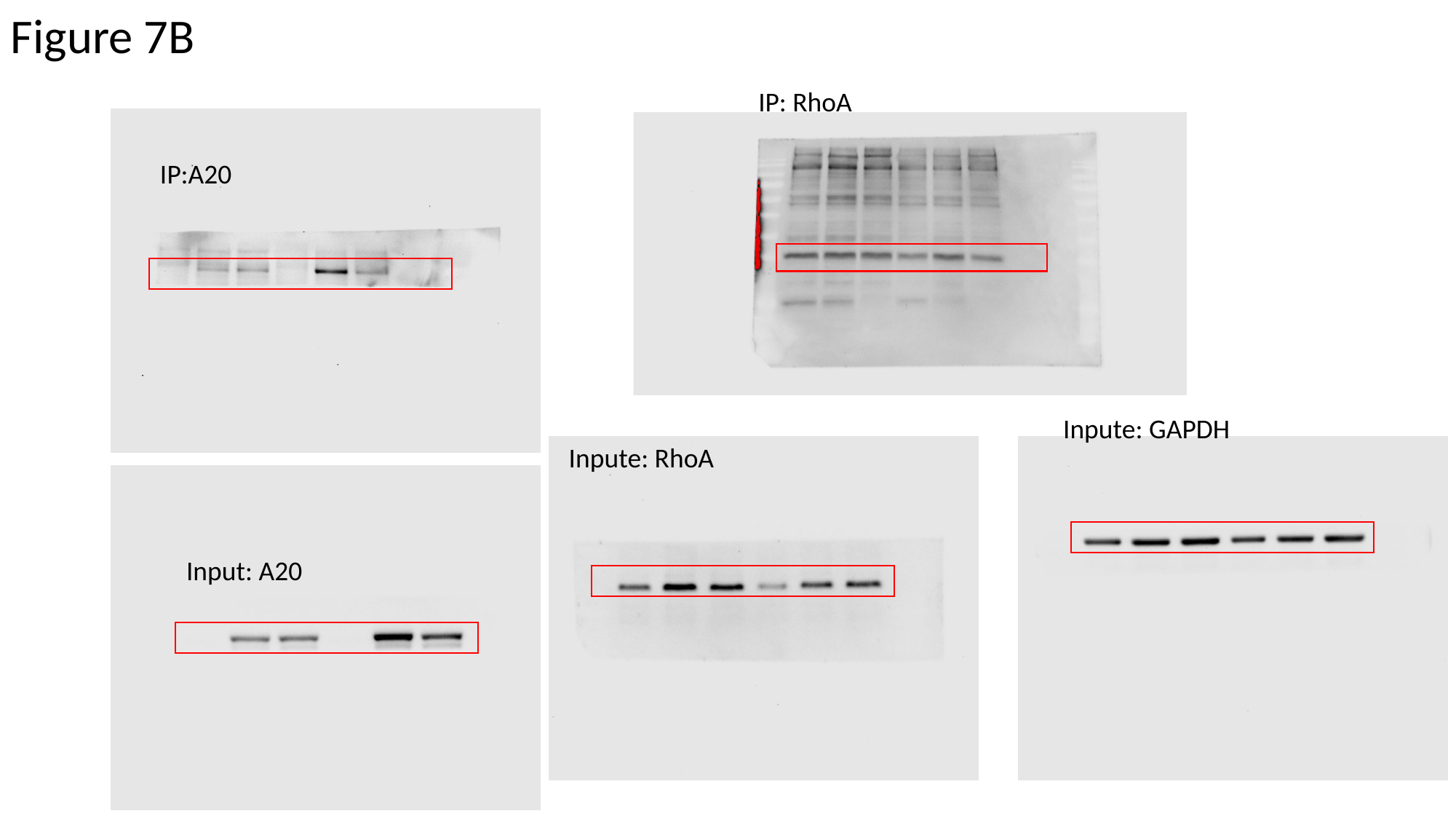

Figure 7B
IP: RhoA
IP:A20
Inpute: GAPDH
Inpute: RhoA
Input: A20

## Slide 6
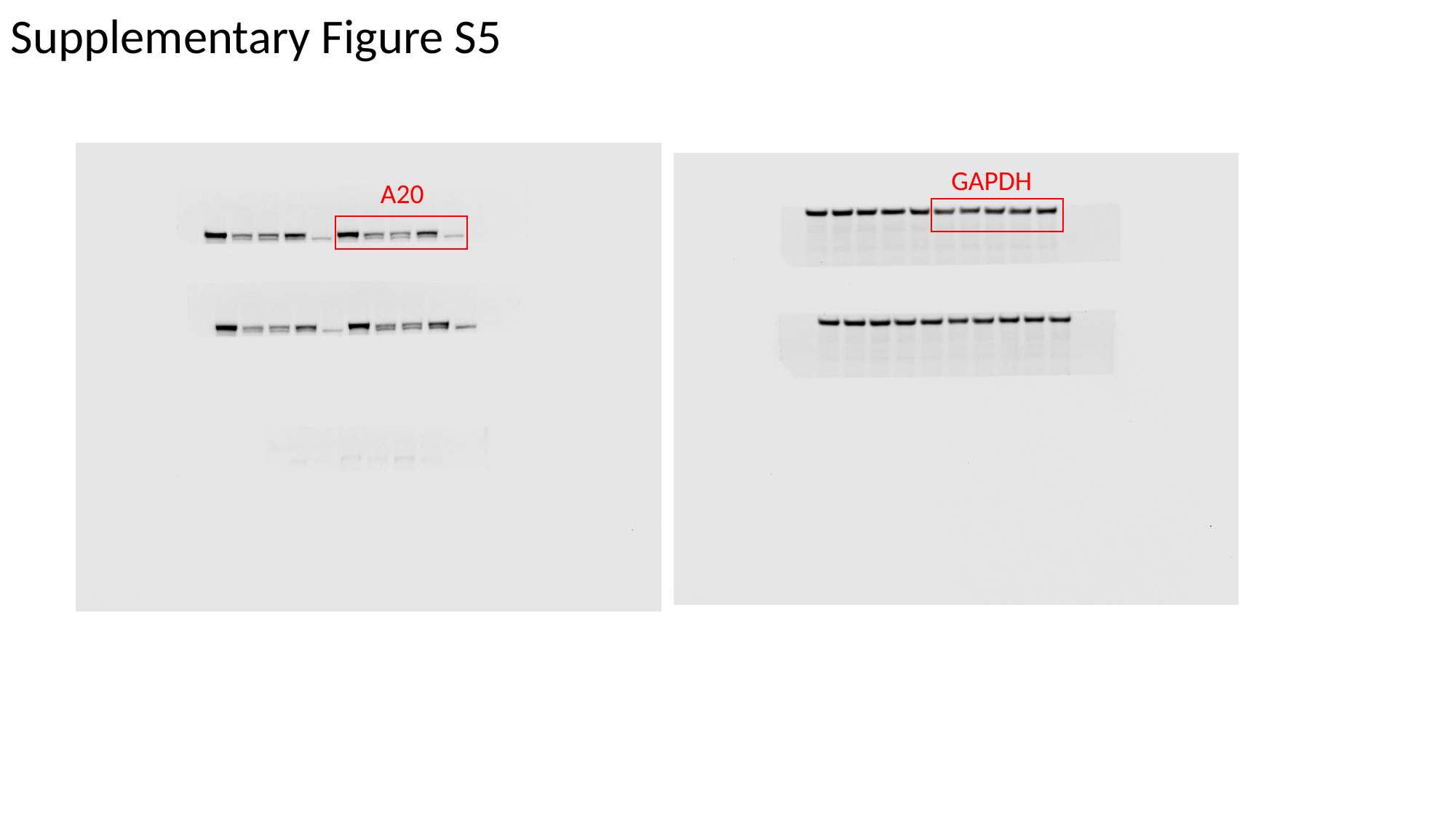

Supplementary Figure S5
GAPDH
A20
